# Controversies, consensuses, and guidelines on macular hole surgery by the Asia–Pacific Vitreo-retina Society (APVRS) and the Asia–Pacific Academy of Professors in Ophthalmology (AAPPO)

**DOI:** 10.1186/s40662-025-00446-0

**Published:** 2025-07-28

**Authors:** Nishant V. Radke, Paisan Ruamviboonsuk, David H. Steel, Tian Tian, Alex P. Hunyor, Andrew S. H. Tsai, Andrew Chang, Chung-May Yang, Chi-Chun Lai, Fangtian Dong, Jennifer I. Lim, Jay Chhablani, Kenny H. W. Lai, Mahesh P. Shanmugam, Peter Stalmans, Pradeep Venkatesh, Robert F. Lam, Se Joon Woo, Shaochong Zhang, Taraprasad Das, Timothy Y. Y. Lai, Vinod Kumar, Xin Huang, Zhaotian Zhang, Zhaoyang Wang, Peiquan Zhao, Dennis S. C. Lam

**Affiliations:** 1 The Primasia International Eye Research Institute (PIERI) of The Chinese University of Hong Kong (Shenzhen), Shenzhen, China; 2 Retina Division, Department of Ophthalmology, Faculty of Medicine, Rajvithi Hospital, Rangsit University, Bangkok, Thailand; 3 Sunderland Eye Infirmary, Sunderland, UK; 4 Biosciences Institute, Newcastle University, Newcastle upon Tyne, UK; 5 Department of Ophthalmology, Xin Hua Hospital Affiliated to Shanghai Jiao Tong University School of Medicine, Kongjiang Road, No. 1665, Shanghai, 200092 China; 6 Macula Research Group, Save Sight Institute, The University of Sydney, Sydney, Australia; 7 Singapore National Eye Centre, Singapore Eye Research Institute, Singapore, Singapore; 8 Duke-NUS Medical School, Singapore, Singapore; 9 Sydney Retina Clinic, Sydney Eye Hospital, University of Sydney, Sydney, NSW Australia; 10 Department of Ophthalmology, National Taiwan University Hospital, Taipei, Taiwan, China; 11 Department of Ophthalmology, College of Medicine, National Taiwan University, Taipei, Taiwan, China; 12 Department of Ophthalmology, Chang Gung Memorial Hospital, Linkou Main Branch, Taoyuan, Taiwan, China; 13 Department of Ophthalmology, Chang Gung Memorial Hospital, Keelung, Taiwan, China; 14 Department of Ophthalmology, State Key Laboratory of Complex Severe and Rare Diseases, Peking Union Medical College Hospital, Key Laboratory of Ocular Fundus Diseases, Chinese Academy of Medical Sciences, Beijing, China; 15 Department of Ophthalmology and Visual Sciences, University of Illinois Chicago, Chicago, IL USA; 16 University of Pittsburgh, School of Medicine, Pittsburgh, PA USA; 17 The C-MER Dennis Lam and Partners Eye Center, C-MER International Eye Care Group, Hong Kong, China; 18 Department of Ophthalmology and Visual Sciences, The Chinese University of Hong Kong, Hong Kong, China; 19 Vitreoretinal and Oncology Services, Sankara Eye Hospital, Bengaluru, Karnataka India; 20 Department Ophthalmology, UZ Leuven, Louvain, Belgium; 21 Department of Ophthalmology, Dr. Rajendra Prasad Centre for Ophthalmic Sciences, All India Institute of Medical Sciences, New Delhi, India; 22 Department of Ophthalmology, Caritas Medical Centre, Hong Kong, China; 23 Department of Ophthalmology, Seoul National University Bundang Hospital, Seoul National University College of Medicine, Seongnam, Republic of Korea; 24 Shenzhen Eye Hospital, Shenzhen Eye Institute, Jinan University, Shenzhen, Guangdong China; 25 Department of Ophthalmology, LV Prasad Eye Institute, Kalam Anji Reddy Campus, Anant Bajaj Retina Institute, Srimati Kanuri Santhmma Centre for Vitreoretinal Diseases, Hyderabad, Telangana India; 26 Eye Institute and Department of Ophthalmology, Eye & ENT Hospital, Fudan University, Shanghai, China; 27 NHC Key Laboratory of Myopia and Related Eye Diseases; Key Laboratory of Myopia and Related Eye Diseases, Chinese Academy of Medical Sciences, Shanghai, China; 28 Shanghai Key Laboratory of Visual Impairment and Restoration, Shanghai, China; 29 State Key Laboratory of Ophthalmology, Zhongshan Ophthalmic Center, Guangdong Provincial Key Laboratory of Ophthalmology and Visual Science, Sun Yat-Sen University, Guangzhou, China; 30 Ophthalmology, Beijing Tongren Eye Center, Beijing Key Laboratory of Ophthalmology and Visual Science, Beijing Tongren Hospital, Capital Medical University, Beijing, China

**Keywords:** Controversy, Consensus, AAPPO, APVRS, Macular hole surgery, Diagnosis, Management

## Abstract

Macular hole surgery, primarily pars plana vitrectomy with internal limiting membrane peeling (ILM) and gas tamponade, has become the standard of care for full-thickness macular hole (FTMH). Despite the 85% to 95% anatomical closure rate, several aspects of the procedure are well accepted whereas some may remain controversial among vitreoretinal surgeons. An international panel of experts (IPE) comprising 27 experts from 10 countries/territories was established to evaluate a total of 38 consensus statements on ILM peeling extent, vital dye selection, face-down positioning requirements, tamponade options, timing of surgery and re-surgery, management of difficult and refractory cases, and adjuvant therapies. The objective is to synthesize evidence-based real-world practice recommendations from leading global experts to guide the management of FTMH. Of the 38 statements, the IPE reached consensus (75% voted as “Strong Agreement” or “Agreement”) on 29 (76.3%). The IPE emphasized the importance of individualized patient factors—such as hole size, chronicity, lens status, and preoperative visual acuity—in surgical planning and tempering patient’s postoperative expectations. There was strong agreement on the need of adequate peeling of the ILM, adjunctive measures including the inverted ILM flap, and face-down positioning for large and refractory FTMH. Controversial statements, such as the use of air tamponade or observation of small FTMH, not reaching consensus are identified. We hope the consensus statements agreed and disagreed by the IPE would help serve as good reference and guidelines in managing FTMH.

## Background

A full-thickness macular hole (FTMH) is a complete defect in the retina at the macula. The condition once deemed untreatable has undergone a remarkable revolution in treatment and strategies. The classification of macular holes (MHs) by size is crucial in determining surgical options and predicting outcomes notably since size serves as a prognostic factor. FTMHs are generally classified into three primary categories based on their minimum diameter: small (< 250 μm), medium (250 to 400 μm), and large (> 400 μm) [1]. This classification allows surgeons to tailor the management approach depending on the size and associated characteristics of the MH [1].

Literature suggests that small MHs have higher spontaneous closure rates than larger ones. It has been observed that the anatomical and visual prognoses following surgical repair are closely correlated with initial hole size—the smaller the hole, the better the surgical outcome [2, 3], and the rate of closure even without surgical intervention is high. For instance, a study indicated that the chance of closure is more substantial in holes < 200 μm, particularly in the absence of vitreomacular traction (VMT) [4]. Non-surgical MH closure using enzymatic vitreolysis has shown to be successful in small MHs [5]. It is to be noted however that the agent for enzymatic vitreolysis—Ocriplasmin is no longer available in the European Union, including United Kingdom [6]. It remains Food and Drug Administration (FDA)-approved for use in MH less than 400 μm but its use has declined over the years due to lower success rates compared to conventional vitrectomy and potential adverse effects like troublesome floaters, photopsia, transient blurring of vision, and pain [7]. These findings underscore the importance of size as a prognostic indicator.

In terms of surgical technique, the literature indicates that larger MHs often necessitate distinct operative strategies in contrast to smaller ones. For example, the inverted internal limiting membrane (ILM) flap technique has gained popularity for larger MHs, as it can enhance closure rates and improve visual outcomes after surgery [8–10]. Studies have shown that the likelihood of successful closure with this technique was high, even in MHs more than 1000 μm [10–12]. The management of larger holes often requires a more complex surgical approach than that of smaller holes, highlighting their different biomechanical and anatomical challenges. A recent study suggested to extend the International Vitreomacular Traction Study (IVTS) classification by defining extra-large MH measuring 550–800 μm in diameter and giant MHs > 800 μm [13].

The IVTS Group provided a framework for understanding the implications of hole size within the surgical context [1]. The classification system delineates cut-off points that inform surgical decision-making, emphasizing that holes greater than 400 μm can often incur worse anatomical and functional surgery results than their smaller counterparts, particularly if the duration of the surgery is long [14]. However, it has to be noted that at the time of the classification, surgery using the inverted ILM flap was not yet published. Studies that quantify preoperative hole size often find that anatomical success diminishes significantly for larger holes, leading to variations in the expected visual acuity outcomes [8, 9, 15].

Multiple analyses of surgical outcomes demonstrate that preoperative hole size is an independent predictor of postoperative visual acuity [16]. A retrospective study revealed that larger holes correlated with significant challenges and inferior postoperative results, reinforcing the predictive significance of MH size prior to surgery [17]. These data emphasize the critical need for meticulous preoperative assessment and individualized surgical planning (e.g., preoperative discussion of potential ILM flap or autologous retinal grafts, preserving anterior capsule in case of a combined phaco-vitrectomy to be used as a graft, or amniotic membrane graft preparation) based on the size of the MH.

Optical coherence tomography (OCT) allows for more precise and repeatable assessments and measurement of MH size compared to traditional methods [1, 18–20]. These imaging modalities facilitate the characterization of hole size and morphology, which are essential for treatment planning and informing the surgeon about the potential success of different intervention methods. Studies involving OCT demonstrated that precise quantification of MH morphometry could predict closure success rates and patient recovery paths more accurately than visual assessments alone [18, 21–23].

Despite a high level of anatomical and functional success achieved, in small or mid-sized FTMH; large FTMH or eyes predisposed to failure such as chronic MH, MH with high myopia, present a particular challenge to the vitreoretinal specialist. Particularly in the Asia–Pacific region where high myopia is more prevalent and serves as a risk factor for MH formation and non-closure post-surgery, there is a pressing need to have a consensus on approaching FTMH in various situations to achieve better outcomes.

Given its high prevalence and its propensity for visual morbidity, and the recent developments in our ability to manage FTMH, the Asia–Pacific Vitreo-retina Society (APVRS) and the Academy of Asia–Pacific Professors of Ophthalmology (AAPPO) felt the need for such consensus statements and guidelines for FTMH management, and two of the senior authors (DSCL and PZ) of this manuscript were appointed to coordinate this consensus project. Despite high success rates, several aspects of the procedure remain controversial among vitreoretinal surgeons. This consensus statement aims to synthesize evidence-based real-world practice recommendations from leading global experts to guide diagnosing and managing MH. The key areas of debate in the surgical treatment of MHs included:

1. ILM peeling extent
2. Vital dye selection
3. Face-down positioning requirements
4. Tamponade options
5. Timing of surgery and re-surgery
6. Management of difficult and refractory cases
7. Adjuvant therapies
8. Outcome assessment
9. Emerging technologies

## Methodology

Further to appointing the coordinators, the APVRS and AAPPO invited three more experts (NVR, PR and TT) to join as core group members. Only English articles from PubMed were included for referencing and cross-referencing. Search was carried out on PubMed using the following terminologies: macular hole, pars plana vitrectomy, ILM peeling extent, vital dyes, face-down position, prone position, endotamponade, endotamponade agents, timing of surgery, re-operation, management, refractory macular hole, adjuvant therapy, adjuvant treatment, success rate, failure rate, and emerging treatments. Initially, Abstracts were screened then full texts of relevant articles were studied to identify areas of consensus and controversy. Any cross references from these articles which were relevant were also screened and used wherever relevant. An indexing-referencing software was used to add references during manuscript preparation. Duplicates entered in the indexing-referencing software were screened using the software and merged to reduce redundancy. The primary focus was on IMH, but refractory MH and special conditions of high myopia, traumatic MH, pediatric MH were also reviewed in brief to have a broader view of existing consensus and controversies in MH management in general. This way the core group of five members performed an extensive literature search and critically reviewed the materials on FTMH, after which the first draft of the manuscript and consensus statements with explanation and elaboration was written. The statements were organized into nine categories as mentioned in the introduction above.

An international panel of experts (IPE) comprising 27 panelists from 10 countries/territories was established. Each panel member independently and anonymously reviewed each statement and provided comments to the core group. The first voting by the IPE members was done using a five-point Likert scale—ranging from strongly agree, agree, neutral, disagree, and strongly disagree. The core group then reviewed and evaluated the feedback and comments, revised it, and sent out the second draft for further opinions and voting. The process was repeated until all the statements were finalized. It took three rounds to have the final list of statements ready. Subsequently, when the final draft was ready, each panel member voted on each statement anonymously. A consensus was reached when at least 75% of the experts voted either ‘agree’ or ‘strongly agree’ for a statement as per the methodology described in a previous consensus paper [24].

## Controversies and consensus statements

### ILM peeling

The extent of the ILM peeling plays a crucial role in the surgical management of IMH. ILM peeling is considered a standard and mandatory procedure in MH surgery, contributing significantly to the anatomical closure rates, which can now exceed 90% with modern techniques [25]. The removal of the ILM is believed to relieve tangential traction that can inhibit the closure of the macular defect, thus enhancing surgical outcomes. Studies indicate substantial improvements not only in anatomic closure but also in visual acuity following ILM peeling as opposed to surgery without peeling [26, 27].

Recent techniques like the inverted ILM flap have been proposed to mitigate the risks of complications, especially in larger MHs [28, 29]. This technique involves rotating the peeled ILM over the hole itself, which also aids in promoting closure and thereby improves visual outcomes in cases that historically had poor prognoses.

Several studies have highlighted the potential complications associated with extensive ILM peeling. Inner retinal defects are commonly observed following ILM peeling and tend to persist once they develop. Studies have discussed concentric macular dark spots as a manifestation of such defects [30, 31]. Similarly, Liu et al. [32], utilizing multimodal OCT imaging, identified inner retinal dimples post-ILM peeling, which corresponded to the observed dark spots. Beyond the mechanical trauma inflicted on the inner retinal layers during ILM removal, Tadayoni et al. [33] demonstrated a significant reduction in retinal sensitivity and a higher incidence of micro-scotomata, emphasizing the importance of limiting not just ILM peeling itself but also the extent of ILM peeling where feasible. Terasaki et al. further noted a decrease in the amplitude of the focal ERG b-wave accompanied by delayed implicit time shortly after ILM peeling [34]. Additional reports have described the development of paracentral scotomas, which are hypothesized to result from nerve fiber layer injury during the procedure [35, 36].

Adding to this complexity, the size of the MH has been shown to influence the necessity and extent of ILM peeling. Large holes may indeed benefit more acutely from partial peeling techniques or inverted flap methods as complete removal could lead to increased postoperative complications without significantly boosting closure rates or affecting final visual outcomes in the long term [37, 38]. Different extents of ILM peeling have been studied by different authors. The ILM peel study group recently showed in an individual participant data (IPD) meta-analysis of five randomized controlled trials (RCTs) and 370 eyes that peeling internal ILM with a peel radius of < 1 disc diameter (DD) in extent may slightly improve the chances of closing an idiopathic FTMH, especially if the hole is larger than 400 µm. However, this had little or no impact on vision after surgery [39]. Bae et al. showed an improvement in postoperative metamorphopsia with a larger extent of ILM peeling (3 DD) compared to that of the group with a smaller extent (1.5 DD) [40]. Khodabande et al. and Yao et al. used 2 DD and 4 DD diameters of areas of ILM peeling to study MH closure rates and found that when macular hole index (MHI) and macular hole closure index (MHCI) were < 0.5 larger extent of peeling resulted in better closure rates without much difference in visual outcomes in the two groups [41, 42]. MHI was first described by Kusuhara et al. and is calculated by dividing the hole height (b) by the basal diameter (a); MHI = b/a [43]. A value > 0.5 was found to be associated with better visual outcomes and chances of closure; i.e., taller hole relative to the base had a better anatomical success [43]. The MHCI is calculated by dividing the of sum of the detached photoreceptor arm lengths (M + N) by the basal diameter (BASE) and a higher MHCI suggests more photoreceptors available to bridge the hole, and thus higher likelihood of “good” or bridge-type closure [21].

These observations reinforce the importance of tailored surgical approaches based on individual patient anatomy and hole characteristics, underscoring the move towards personalized surgical strategies in contemporary ophthalmology.

Therefore, surgical decision-making often involves balancing the benefits of extensive peeling against the risks of iatrogenic damage. In summary, the extent of ILM peeling is a critical determinant in the management of IMHs, with contemporary techniques evolving to maximize surgical success while minimizing associated risks.

***Consensus Statement 1.1:*** *ILM peeling is considered a standard procedure in MH surgery. [Consensus score: 100% (strongly agree: 92.59%; agree: 7.41%)].*

***Consensus Statement 1.2:*** *The extent of the ILM peeling plays a crucial role in the surgical management of MHs, especially for holes larger than 400 μm. [Consensus score: 96.3% (strongly agree: 66.67%; agree: 29.63%)].*

***Consensus Statement 1.3:**** For larger MHs and MHs with the MHI or MHCI* < *0.5, a 4-disc area of peeling centered on the MH would be safer for achieving anatomical success. [Consensus score: 88.89% (strongly agree: 37.04%; agree: 51.85%)].*

***Consensus Statement 1.4*** *: Functional outcomes can be marred by micro-scotomata, reduced retinal sensitivity, and decreased vision (although not that common) despite successful structural outcomes and hence judicious, careful and atraumatic peeling of ILM is essential. [Consensus score: 92.6% (strongly agree: 55.56%; agree: 37.04%)].*

### Vital dye selection

Brilliant blue G (BBG), when compared with other agents such as indocyanine green (ICG) and trypan blue, has demonstrated a lower incidence of retinal toxicity compared to ICG and effective staining capabilities compared to trypan blue. It allows for clear visualization of the ILM, enabling surgeons to differentiate between peeled and unpeeled areas easily [44, 45]. The optimal duration of dye contact with the macula for effective staining without toxicity is typically around 30 s to 1 min; this timeframe balances efficient staining with the minimization of potential phototoxic effects [45]. Some studies have used a duration as short as 5 s to achieve visible staining with BBG [46]. Since failed MHs are also presented to the retina clinic, re-surgery entails restaining the macula with vital dyes. Sometimes, restaining the macula is needed during primary surgery. At present, there are no definite guidelines on the duration of restaining such retinae. Intraoperative OCT may be helpful in such cases to avoid possible drawbacks of staining the retina including a part denuded of ILM though this equipment is not universally available.

Reducing light intensity during macular manipulation is essential for safeguarding retinal health, particularly when using intraoperative dyes. Higher light exposure can exacerbate the phototoxic effects associated with dyes like ICG, which has been shown to cause damage to retinal structures if concentrations and exposure times are not carefully controlled [47]. Hence, using lower illumination levels during critical phases of surgery helps decrease the likelihood of postoperative visual disturbances. In this context, tailoring light intensity not only aids in preserving retinal integrity but also enhances overall surgical safety, ultimately contributing to improved visual outcomes after surgery. The understanding of dye contact duration is crucial as it affects both the efficiency of the staining process and the overall safety of the surgical procedure. Some surgeons prefer to stain the ILM under air while some surgeons use dyes with an integrated carrier like polyethylene glycol which can be used in a fluid filled eye directly. Prolonged exposure can potentially lead to retinal toxicity, as highlighted in several studies, reinforcing the importance of adhering to these recommended guidelines during surgery [48–50].

In summary, utilizing BBG as a dye, maintaining optimal exposure durations during surgery, and ensuring appropriate light regulation creates a safer surgical environment for patients undergoing ILM peeling for MHs. Studies on restaining may address safety concerns in future.

***Consensus Statement 2.1:*** *BBG is a good choice of dye for ILM staining due to its effective visualization properties compared to trypan blue and lower risk of retinal toxicity compared to alternatives such as ICG and trypan blue. [Consensus score: 88.89% (strongly agree: 48.15%; agree: 40.74%)].*

***Consensus Statement 2.2:*** *Minimizing dye contact time to approximately 30 s to 1 min is recommended to ensure sufficient ILM staining while reducing the risk of phototoxic effects on the retina. [Consensus score: 92.6% (strongly agree: 55.56%; agree: 37.04%)].*

***Consensus Statement 2.3:*** *Reducing intraoperative light intensity to reduce the photo-chemical interaction and toxicity during dye-assisted procedures is essential to prevent phototoxic damage and optimize retinal safety, especially when using dyes with higher toxicity profiles such as ICG. [Consensus score: 92.6% (strongly agree: 55.56%; agree: 37.04%)].*

### Face-down positioning

The role of face-down or prone positioning after surgery for refractory and large FTMH is important for achieving successful closure rates. This position is intended to utilize gas tamponade to maintain the closure of the MH by ensuring the gas bubble exerts pressure on the hole while keeping it in contact with the retinal pigment epithelium and thus maintain dry edges which are essential for closure.

Several studies highlight that maintaining face-down positioning significantly impacts surgical success. Studies indicate that patients positioned face-down experienced improved closure rates for MHs larger than 400 µm [51]. The face-down positioning study group in an individual participant meta-analysis of five RCTs with 379 eyes reported low certainty evidence of benefits of face-down positioning in MH sizes < 400 µm pending further investigations; for MH > 400 µm, face-down positioning has been recommended [52]. Further, the Positioning in Macular Hole Surgery (PIMS) trial compared face-down with face-forward positioning for 8 h daily over 5 days. The findings suggest that face-down positioning may not provide a definitive benefit in improving outcomes for large MHs, raising questions about the necessity of recommending this posture following surgery [53].

Recent comparative studies have reported similar anatomical success rates among patients with varying postoperative positioning strategies, suggesting that optimal positioning may depend on individual patient needs [54, 55]. Thus, while traditional practices emphasize face-down positioning, evolving evidence suggest that flexibility in postoperative positioning may still achieve satisfactory outcomes for MH repair. It is difficult to get good images using spectral-domain OCT (SD-OCT) in the immediate postoperative stage due to the presence of intraocular gas and sometimes due to coexisting cataracts. In such cases, swept-source OCT (SS-OCT) has demonstrated the ability to image the macula as early as 24 h after MH surgery. Cases which had a successful closure were advised to stop face-down position and their holes did not open up in the follow-up [56]. This approach can help minimize patient discomfort and customize advice for patients. Several studies suggest that shorter durations of prone positioning, ranging from one night to 3 days, can be sufficient for successful anatomical closure of MHs, especially when ILM peeling and gas tamponade are used. For instance, Isomae et al. reported comparable closure rates (~90%) with just 1 day of prone posturing compared to 1 week [57]. Krohn demonstrated that 3 days of posturing yielded success rates similar to seven days (87.5% vs. 93.1%) [58], while Almeida et al. reported a 98% closure rate with 3-day prone positioning [59]. A pilot study found that 5 days of prone posturing for at least 12 h per day also produced favorable outcomes, although many patients struggled with adherence [60]. These findings collectively suggest that strict and prolonged prone positioning (e.g., 7 days) may not be necessary for all patients, and shorter, more tolerable regimens (1–3 days, or even just one night) may offer similar anatomical results in selected cases, particularly for smaller or less chronic MHs.

***Consensus Statement 3.1:*** *Face-down positioning remains a widely used postoperative strategy, particularly in cases of large or refractory MHs, to enhance gas tamponade efficacy and promote anatomical closure. [Consensus score: 88.89% (strongly agree: 59.26%; agree: 29.63%)].*

***Consensus Statement 3.2:*** *Emerging evidence suggests that successful MH closure may still be achieved without strict adherence to face-down positioning, especially when intraocular gas tamponade is sufficient and tailored to patient-specific factors. [Consensus score: 85.18% (strongly agree: 40.74%; agree: 44.44%)].*

***Consensus Statement 3.3:*** *Early postoperative imaging with SS-OCT may allow for individualized guidance on face-down positioning, potentially reducing patient burden without compromising surgical outcomes* ***.*** *[Consensus score: 85.18% (strongly agree: 33.33%; agree: 51.85%)].*

### Tamponade selection

In the management of IMHs, the selection of endotamponade agents is critical for ensuring optimal surgical outcomes. Choices often include sulfur hexafluoride (SF6) gas, hexafluoroethane (C2F6) gas, air, and silicone oil. Each option presents specific advantages and disadvantages, necessitating careful deliberation concerning patient circumstances and desired outcomes (Table 1).

**Table 1:** Comparative overview of tamponade agents used in macular hole surgery

Tamponade agent	Advantages	Limitations
Sulfur hexafluoride (SF6)	Rapid absorption, typically within 1–2 weeks, allowing for quicker postoperative recovery and less dependence on prolonged face-down positioning It is considered effective in routine cases, with reported anatomical closure rates of approximately 90% [54, 55]	Due to its short duration of tamponade, SF6 may not provide sufficient support in cases requiring prolonged closure, potentially compromising surgical outcomes
Perfluoro propane (C3F8)	Longer duration of tamponade duration, approximately 2–3 weeks which is particularly advantageous in complex cases [69] This prolonged effect has been associated with improved closure rates in large macular holes (MHs) [69]	Prolonged visual recovery. Risk of cataract progression with its use [70]
Air tamponade	Rapidly absorbed, early recovery and less prolonged postoperative positioning [69, 71]. Cost-effective and logistically simpler compared to gas	Reduced efficacy in larger or more complex MHs. Lower closure rates and an increased need for reoperations in larger MH [71, 72]
Silicone oil	Suitable for recurrent or complex MHs, particularly those associated with retinal detachment [73–75] Relaxed face-down positioning, improving patient comfort and compliance	A secondary procedure to remove the oil, introducing additional surgical risks Risks of elevated intraocular pressure, cataract, oil emulsification, epiretinal membranes formation

While Kelly and Wendel [61] initially used SF6 gas for tamponade, many surgeons later favored long-acting gases such as perfluoropropane (C3F8) to prolong gas contact and enhance MH closure. Silicone oil has also been employed for similar reasons. However, current practice has shifted toward medium-acting (C2F6), short-acting (SF6) gases, and even air. Concurrently, the necessity of face-down positioning has been questioned, especially when longer-acting gases like C2F6 and C3F8 are used. A recent RCT found less than a 10% improvement in closure rates with 5 days of face-down positioning vs. face-forward positioning in large holes (> 400 µm) using C3F8 [53].

The relationship between tamponade type and positioning likely reflects the duration needed for effective hole bridging. If gas fill exceeds 50%, even an upright posture may suffice [62]. The exact time required for tamponade-induced closure remains uncertain, as some holes close spontaneously or via enzymatic vitreous detachment without tamponade [63]. OCT-based studies show most closures occur by day two to three postoperatively, with minimal reopening by three months [64]. Hole size and chronicity significantly affect closure potential and guide tamponade selection [65–67]. Variability in study designs, including posture protocols and ILM peeling, complicates interpretation of the existing evidence [68].

The choice of tamponades in MH surgery remains a complex decision influenced by factors such as MH complexity, individual patient needs, and potential postoperative implications. SF6 and C3F8 have their respective advantages regarding closure rates and recovery times, while air presents a practical option for simpler cases despite its lower success rates. It should be noted that C2F6 and C3F8 are Per- and polyfluoroalkyl substance (PFAS) molecules, hence they are at risk of being included in a total ban of fluorinated molecules in the European Union by 2027. SF6 is not a PFAS, but a potent greenhouse gas which unfortunately is also far from environmentally friendly. Silicone oil therefore, stands out as a robust choice in challenging scenarios but comes with its own set of complications and the need for subsequent surgical intervention. A tailored approach that considers these variables is instrumental in achieving optimal surgical outcomes.

***Consensus Statement 4.1:*** *The selection of endotamponade agents in MH surgery should be personalized based on hole size, chronicity, and patient-specific factors. [Consensus score: 100% (strongly agree: 66.67%; agree: 33.33%)].*

***Consensus Statement 4.2:*** *SF6 gas is an effective tamponade option in routine IMH surgeries, balancing closure rates and recovery time. [Consensus score: 81.48% (strongly agree: 40.74%; agree: 40.74%)].*

***Consensus Statement 4.3:*** *Long-acting gases such as C3F8 improve anatomical success in large MHs but increase patient burden due to prolonged face-down positioning and cataract risk [Consensus score: 92.59% (strongly agree: 51.85%; agree: 40.74%)].*

***Consensus Statement 4.4:*** *Air tamponade is a viable choice for small or uncomplicated MHs, despite reduced success rates in larger or chronic cases. [Consensus score: 70.37% (strongly agree: 37.04%; agree: 33.33%)].*

***Consensus Statement 4.5:*** *Silicone oil tamponade should be reserved for recurrent or complex MHs, due consideration to its long-term complications and the need for a second surgery. [Consensus score: 88.89% (strongly agree: 48.15%; agree: 40.74%)].*

### Surgical timing and indications

The timing of surgical intervention for IMH remains a topic of ongoing debate and diverse clinical approaches. Scientific literature provides insights into whether immediate surgery or an observation period is preferable, influenced by factors such as hole size and duration, presence of VMT, potential for spontaneous closure, success rate of the surgery and patient’s willingness to accept the surgery, etc.

#### Immediate vs. delayed intervention

##### Immediate surgery

Proponents of immediate surgical intervention advocate for early vitrectomy as it maximizes chances for successful outcomes. Immediate treatment is especially encouraged in cases with larger MHs. Early intervention typically leads to better anatomical success rates and potentially improved visual outcomes [76, 77]. Some studies suggest that delaying surgery for patients with large holes or significant VMT may result in degeneration of the foveal architecture, complicating the surgical repair [77].

##### Delayed surgery

Conversely, proponents of a delayed approach argue for initial observation for small MHs. The rationale for observation is supported by reports indicating that 0% to 15.8% of small, asymptomatic holes (< 250 μm) may close spontaneously over time [78, 79]. Using tools such as OCT, the status of the hole can be monitored, allowing for timely intervention should it progress [78, 80]. Traumatic MHs often are subjected to delayed surgery because of potential for spontaneous closure [81].

#### Observation period for small holes

The observation period for small MHs is often characterized by regular follow-ups. A median duration of 44 days for spontaneous closure was found in a study on small MHs [78]. However, progressive symptoms such as visual acuity decline can warrant earlier surgical action.

#### Role of VMT in decision-making

VMT plays a critical role in determining the timing for surgical intervention. In cases where VMT is present, surgery is often preferred due to the associated risk of the hole progression. Research has shown that VMT can exacerbate retinal damage and delay closure [77]. A recently published study shows that pneumatic release of VMT can successfully close MHs omitting the need for surgery [82]. Such decision-making underscores the importance of comprehensive imaging and clinical assessment in crafting a patient-specific surgical timeline.

#### Optimal timing for maximal visual outcomes

The optimal timing for surgery is also contingent on a variety of factors, including the stage of the MH. Literature outlines enhanced visual outcomes when surgery occurs within three months of symptom onset [83–86]. However, research suggests that significant improvements can still be observed even when surgery is performed later, although this is generally contingent on individual factors, such as hole characteristics [87].

In conclusion, surgical timing for MHs reflects a balance between immediate intervention and an appropriate observation period, factoring in VMT status and hole morphology with a preference for an early intervention (within three months) for a better prognosis. Each case should be individualized based on the severity of symptoms, hole size, and imaging findings, with an active discussion between the patient and ophthalmic surgeon to determine the best course of action for optimal outcomes.

***Consensus Statement 5.1:*** *Early surgical intervention is recommended for larger MHs or those associated with VMT to prevent foveal architectural damage and improve visual outcomes. [Consensus score: 85.18% (strongly agree: 40.74%; agree: 44.44%)].*

***Consensus Statement 5.2:**** Small, asymptomatic MHs (*< *250 µm) may be managed conservatively with an initial observation period, provided regular OCT monitoring is in place. [Consensus score: 62.96% (strongly agree: 33.33%; agree: 29.63%)].*

***Consensus Statement 5.3:*** *The presence of VMT is a strong indication for early surgery due to its association with hole progression and retinal damage. [Consensus score: 77.78% (strongly agree: 29.63%; agree: 48.15%)].*

***Consensus Statement 5.4:*** *Visual outcomes are generally better when MH surgery is performed within three months of symptom onset, though individualized timing may still yield favorable results. In general, earlier surgery is recommended. [Consensus score: 100% (strongly agree: 59.26%; agree: 40.74%)].*

***Consensus Statement 5.5:**** The decision between immediate surgery and delayed surgery should be individualized, taking into account characteristics of the hole, success rate and patient’s visual need and willingness to accept the surgery, *etc*. [Consensus score: 92.59% (strongly agree: 59.26%; agree: 33.33%)].*

### Management of special cases

The management and resolution of FTMH continue to be critical areas of research due to the challenges posed by recurrent and refractory cases. Understanding the risk factors associated with surgical failure can guide clinicians in optimizing treatment strategies and improving surgical outcomes.

#### MH size

The initial size of the MH is a significant predictive factor for surgical success. Majority of studies discuss that larger holes (> 400 µm) have a higher likelihood of surgical failure. However, more recent studies suggest that the change starts happening at 500 µm with the success rate of MH closure dropping from 97% to 90% at 500 µm. Notably, there was no change at 400 µm and perhaps future studies could be directed with this as a cut-off of 500 µm [66]. Maguire an colleagues have reported that a larger hole size correlates with worse anatomical and visual outcomes post-surgery [16].

#### Duration of symptoms and chronicity of MH

The length of time the MH has been present prior to surgical intervention is another critical factor. Holes with duration longer than four months have been reported to have a lower chance of closure as compared to those lesser than four months [66]. Prolonged duration is associated with increased failure rates as chronic holes exhibit more complex anatomical and structural changes, making closure more difficult [3, 16]. Chronic FTMH is typically more challenging to repair than acute cases, largely due to retinal atrophy and scarring that can occur over time [88]. The chronic nature of the holes often results in loss of inherent retinal elasticity that impedes successful closure post-surgery.

#### Vitreomacular interface, vitreofoveal attachment and presence of ERM

The attachment of the vitreous to the fovea may promote tangential traction contributing to the formation and persistence of MHs. Chun et al. discussed the role of vitreofoveal attachments in the progression of MHs in the fellow eyes of MH patients [89]. The involvement of an epiretinal membrane (ERM) spanning the MH margin has been found to markedly increase the chances of surgical failure. Research indicates that eyes with both marginal ERM and epiretinal proliferation are at a significantly higher risk of unfavorable outcomes post-surgery [90]. Higher preoperative tractional forces can complicate surgical repair. Studies have pointed to VMT as a significant risk factor, potentially leading to hole formation [1].

#### Myopia

High myopia is associated with various vitreoretinal pathologies [91, 92], making these patients more susceptible to developing MHs. Myopia complicates the surgical landscape by increasing the risk of failing to achieve adequate closure during the procedure [93–95].

#### Re-operation history, patient age and comorbidities, and preoperative patient vision

Eyes with a previous history of failed MH surgery may have reduced chances of success upon subsequent interventions, as shown in studies highlighting the diminished closure rates in reoperations [16]. Advanced age often correlates with poorer healing capacities. Comorbidities such as diabetes or hypertension could further complicate the outcomes of macular surgeries by potentially affecting healing and recovery postoperatively. Preoperative visual acuity often serves as a prognostic indicator for post-surgical outcomes. Eyes with poor vision prior to surgery may have diminished potential for recovery or successful closure as compared to eyes with better preoperative vision [66].

#### Pediatric MHs

These are less common than their adult counterparts and primarily result from trauma or congenital conditions, such as Coats' disease, rather than aging processes prevalent in adults [96, 97]. Surgical intervention using vitrectomy and ILM peeling in children has demonstrated high closure rates, with some reports indicating closure rates of around 92% in selected cases [97].

Management strategies often involve unique considerations, as children's eyes are still developing, which may influence both surgical techniques and outcomes. Pediatric patients generally have better regenerative capabilities, contributing to favorable anatomical and functional post-surgery results. However, complications such as retinal detachment may still pose significant risks [96].

While pediatric and adult MHs share surgical strategies, differences in etiology, treatment approaches, and recovery outcomes highlight the importance of tailored management in the pediatric population.

In summary, there are numerous factors that contribute to the risk of surgical failure in cases of FTMHs. Understanding these risk factors—size of the hole, duration of symptoms, structural changes in the retina, patient demographics, prior surgical history, and coexisting retinal issues—can significantly inform treatment approaches and improve prognostic consultations for patients contemplating MH surgery.

***Consensus Statement 6.1:**** MH size* > *400 μm is a strong predictor of surgical failure correlating with both poorer anatomical closure rates and diminished visual outcomes. [Consensus score: 88.88% (strongly agree: 44.44%; agree: 44.44%)].*

***Consensus Statement 6.2:*** *Prolonged duration of symptoms prior to surgery significantly increases the risk of surgical failure, due to retinal atrophy and fibrotic changes associated with chronic FTMH. [Consensus score: 92.59% (strongly agree: 62.96%; agree: 29.63%)].*

***Consensus Statement 6.3:*** *The presence of ERMs and vitreofoveal attachments are key contributors to surgical failure in MH repair, due to increased tangential traction and structural complexity* ***.*** *[Consensus score: 55.55% (strongly agree: 22.22%; agree: 33.33%)].*

***Consensus Statement 6.4:*** *High myopia independently raises the likelihood of unsuccessful MH closure, owing to associated vitreoretinal structural abnormalities. [Consensus score: 100% (strongly agree: 66.67%; agree: 33.33%)].*

***Consensus Statement 6.5:*** *Patient-related factors such as older age, systemic comorbidities (e.g., diabetes, hypertension), prior failed surgery, and poor preoperative visual acuity substantially affect surgical prognosis and visual recovery. While patients with MH in eyes with diabetic retinopathy may have poor outcome, diabetes and hypertension themselves may not be poor prognostic indicators. [Consensus score: 88.89% (strongly agree: 51.85%; agree: 37.04%)].*

### Adjuvant therapies

Large, failed, refractory, chronic and recurrent FTMHs present significant clinical challenges. Over the past two to three decades, advancements in surgical techniques have emerged to address these cases. The ReMaHo study evaluated 116 FTMHs without residual ILM, treated with human amniotic membrane graft (AMG), autologous ILM flap (AILMT), or autologous retinal graft. Overall closure was achieved in 92% of cases. Visual acuity improved significantly across all groups. ART-treated eyes had larger holes and worse final visual outcomes. AMG was more effective than AILMT for holes > 680 μm. Preoperative BCVA correlated with postoperative vision, guiding surgical choice [98]. Thus, although prognosis may be affected by other patient factors, a re-surgery could be contemplated. Some lesser studied graft options include the use of an autologous tenons capsule graft [99] or Descemet membrane epiretinal graft [100]. Since these are not widely studied as yet, the following enumeration describes the common techniques published in relevant literature during this timeframe involving the use of adjuvants. It should however be noted, and also communicated to the patients that, although anatomical success is possible, functional improvements may not always be significantly achieved.

#### Inverted ILM flap and ILM translocation techniques

This technique involves creating a flap from the ILM and positioning it over the MH to promote closure. Studies indicate that this approach enhances anatomical closure rates, particularly in large or persistent holes. A recent large IPD analysis of 13 RCTs and 792 eyes showed that ILM flaps significantly improve closure rates particularly in holes > 500 μm where the number needed to treat is as low as four, with a significant improvement in vision. Variations in adjunctive use of ILM in the form of ILM inversion, ILM graft translocation, Pedicle graft, ILM tuck, and ILM free graft or pedicle graft under perfluorocarbon liquid have been tested successfully [8, 9, 28, 101–105].

#### Non-inverted free flap technique

**Fig. 1:**
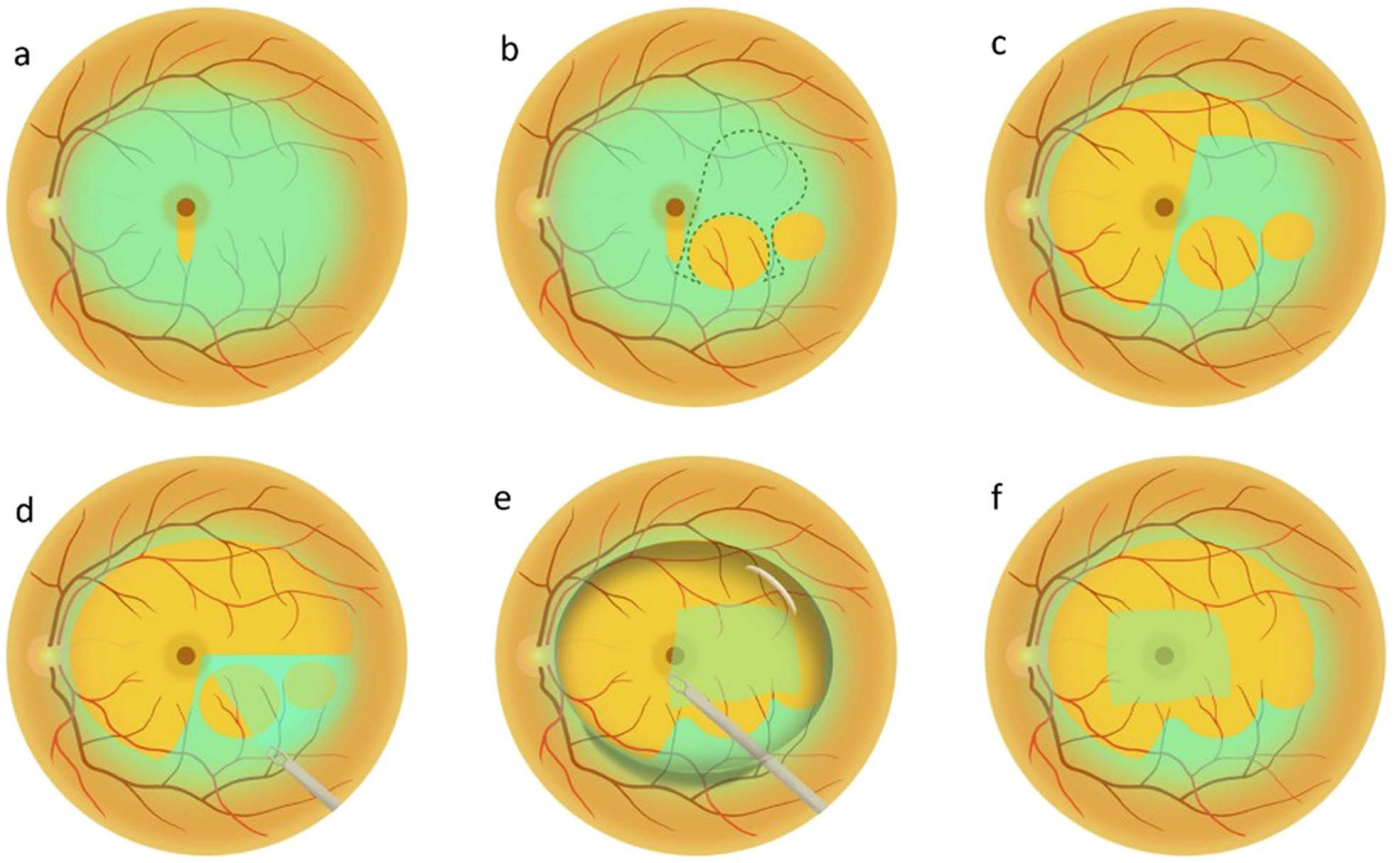
Schematic illustration of the non-inverted “plastic bag” internal limiting membrane (ILM) free flap technique. **a** “Tongue-shaped” peeling. **b** Two “handles” formation. **c** Circular peeling process. **d** Formation of the “plastic bag”: flap with two pedicles. **e**, **f** Perfluorocarbon liquid (PFCL) injection, flap placement and final adjustment. Adapted from “Non-inverted and single-layer “plastic bag” ILM flap novel technique to treat large macular holes” by Zhao et al. 2025, Asia-Pac J Ophthalmol. In press (Creative Commons CC-BY license) [106]

A novel technique, using a non-inverted free ILM flap to treat large MH repair has been proposed by Tian et al. with good initial results (Fig. 1) [106]. The difference primarily with an inverted flap is the ability to retain the retinal side intact. Further studies are warranted to investigate its safety and efficacy.

#### Autologous lens capsule flap transplantation

This innovative method involves transplanting the patient’s lens capsule to promote closure in persistent MHs. Preliminary studies reflect its potential efficacy in cases resistant to conventional surgical techniques or where initial ILM peeling was large enough and no more ILM can be harvested [107, 108]. In young patients with crystalline lens, ILM peeling is easy, and the lens should not be sacrificed in general. However, if any lens opacity precludes ILM visualization and peeling then the cataract should be treated together with the MH surgery. If the lens was preserved in the primary surgery, there is an opportunity to use the lens capsule as a graft material during re-surgery should the need arise. Posterior capsular grafts have also been used in failed or refractory MH [109] however, it is more challenging to harvest the posterior capsular graft than an anterior capsular graft. Hence, the decision to co-treat the lens is often nuanced and individualized to patients’ needs as well as taking into account the surgeon’s comfort from a viewpoint of safety while managing MH.

***Consensus Statement 7.1:*** *The inverted ILM flap and translocation techniques significantly improve anatomical closure rates in persistent or MHs larger than 400 μm. [Consensus score: 92.59% (strongly agree: 62.96%; agree: 29.63%)].*

***Consensus Statement 7.2:*** *It is surgically challenging to maintain free ILM graft over the hole. However, the non-inverted free ILM flap technique shows good results and is worthy of consideration and further studies to confirm its validity are warranted. [Consensus score: 74.08% (strongly agree: 37.04%; agree: 37.04%)].*

***Consensus Statement 7.3:*** *Autologous lens capsule flap transplantation is a viable option when ILM tissue is unavailable for grafting. [Consensus score: 74.08% (strongly agree: 25.93%; agree: 48.15%)].*

#### Amniotic membrane grafting (AMG) and autologous retinal transplantation (ART)

Amniotic membrane grafts have been utilized to promote healing and closure of refractory MHs. The use of amniotic membranes has shown promising results in achieving anatomical closure in patients where traditional methods have failed [110, 111]. ART, a relatively novel technique involves harvesting a graft of the patient's retina and relocating it to the site of the MH. Research indicates that ART can be effective in managing chronic, large, refractory MHs [112–114]. In some complex cases, creating additional retinal flaps (e.g., nasal retinotomy) have been applied to facilitate closure of stubborn MHs. This technique, although more invasive, can yield successful outcomes where simpler methods have failed [115, 116]. The technique being invasive, and the visual outcome has not been encouraging and is best used with caution.

#### Macular buckle

Macular buckle either used alone or in combination with pars plana vitrectomy and ILM peeling with endotamponade has been shown to be effective in achieving the release of myopic macular traction as well as in achieving myopic MH closure in about 70% of patients. The axial length reduction achieved decreases the risk of worsening atrophic myopic maculopathy besides addressing the anteroposterior traction more completely [117–120]. It is to be noted however that at the time or writing there is no commercially available macular buckle which is FDA approved or bears the Conformite Europeenne mark.

***Consensus Statement 7.4:*** *AMG is an option to be resorted to if ILM techniques have failed and lens capsular graft harvesting is not possible. They can anatomically close MHs but often yield suboptimal visual outcomes. Due to limited functional recovery, these techniques are reserved as last-resort options when conventional surgeries fail to achieve hole closure. [Consensus score: 96.3% (strongly agree: 37.04%; agree: 59.26%)].*

***Consensus Statement 7.5:*** *ART is an option to be resorted to if ILM techniques have failed and lens capsular graft harvesting is not possible. They can anatomically close MHs but often yield suboptimal visual outcomes. Due to limited functional recovery, these techniques are reserved as last-resort options when conventional surgeries fail to achieve hole closure. [Consensus score: 88.88% (strongly agree: 44.44%; agree: 44.44%)].*

***Consensus Statement 7.6:*** *Macular buckle surgery can be an effective strategy, particularly in myopic traction maculopathy or cases requiring axial length reduction. Further studies are warranted. [Consensus score: 81.48% (strongly agree: 33.33%; agree: 48.15%)].*

#### Light intensity laser burn to the base of the MH

##### Laser to the base of MH

**Fig. 2:**
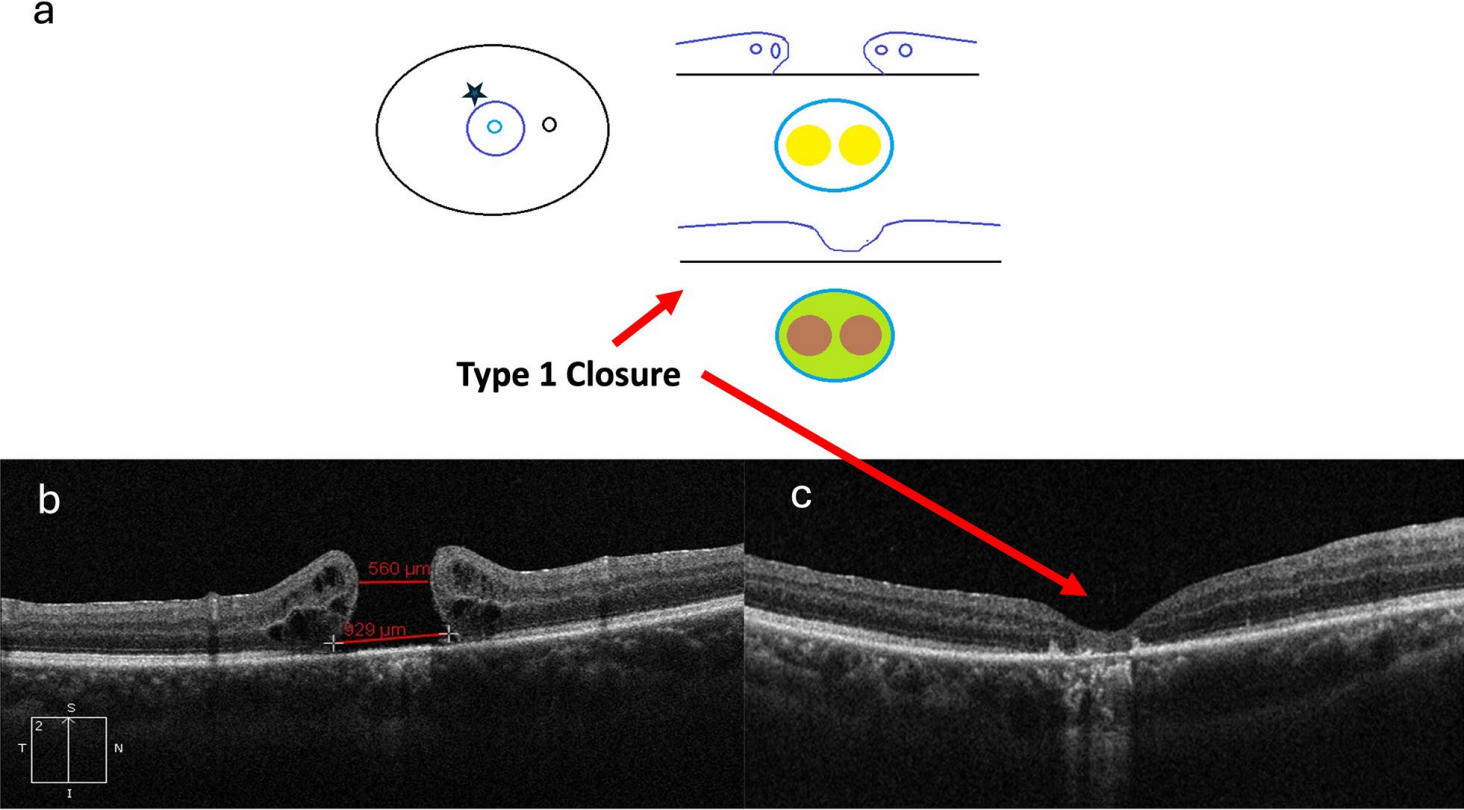
Schematic drawing of the laser application at the base of the macula hole, and the preoperative and postoperative optical coherence tomography (OCT) photos. **a** The schematic drawing showing the edges of the macular hole (MH) in blue circle. The two yellow spots represented the light tissue reaction resulting from the light-intensity laser burn right after the surgery. The two brownish spots represented some mild tissue reaction after the MH was closed. **b** Preoperative OCT showing full-thickness MH. **c** Postoperative OCT showing type 1 closure of the MH

Highly myopic eyes with MHs pose a particular challenge since they have a high chance of failure. A simple technique of applying low-intensity burns within the MH crater to the retinal pigment epithelium has been shown to promote gliosis and achieve closure of MH in pathological myopia eyes with MH-retinal detachment and long axial lengths. This method when combined with 15% C3F8, can result in good anatomical closure and functional improvement in MHs in the size range of 400–800 µm or MH with a high risk of failure. Radke et al. in their paper on MH also discussed the technique of applying two or sometimes three low-intensity (60–100 mW; 100 mS) directly within the MH, taking care to avoid a retinal tissue burn (see Fig. 2) [121]. This remains a simple tool in the armamentarium and can be used when adjuvant techniques have been tried and failed; however, further studies are warranted.

***Consensus Statement 7.7:*** *Low power and mildly intense laser burn applied at the base of the MH can be considered as an easy and less manipulative method to achieve successful closure of high myopic MHs or MHs ranging from 400 to 800 µm. [Consensus score: 33.33% (strongly agree: 18.52%; agree: 14.81%)].*

#### Spontaneous closure observation

Lastly, the spontaneous closure of MHs, particularly chronic ones, has been noted in certain cases, suggesting the necessity of careful monitoring before resorting to further surgical options [122, 123].

In conclusion, the landscape for managing failed, refractory, and recurrent FTMHs has evolved significantly over the past couple of decades. A combination of innovative surgical techniques, adjunctive therapies, and tailored individualized treatment plans is crucial for improving outcomes for patients who have undergone prior unsuccessful surgeries.

### Visual outcomes and recovery

Current literatures suggest that surgical intervention for FTMHs can be challenging, particularly when previous surgeries have resulted in failure. A multicenter study highlighted that revision surgery for FTMH often has varying anatomical closure rates depending on several factors, including the initial size of the hole, the presence of ERMs, and the duration of the hole prior to surgical intervention [16]. A large sample study has shown that the presence of ERMs during re-surgery complicates outcomes as they can inhibit the healing process and increase the likelihood of further surgical failure [90]. The authors concluded that ERM around the hole margins or ERM proliferation were both likely to result in failure as compared to eyes without ERM [90].

In cases where primary surgical efforts have failed, adjunctive therapies, have been investigated as potential adjunct therapies to mitigate the risk of recurrence [16]. This approach underscores the evolving paradigm in the management of complex cases where traditional methods may not yield favorable results.

***Consensus Statement 8.1:*** *Visual improvement after anatomical closure in revision FTMH surgeries is not guaranteed. However, vision does improve in most cases after anatomical closure though improvement may be suboptimal and quite depends on preoperative retinal integrity. [Consensus score: 96.29% (strongly agree: 62.96%; agree: 33.33%)].*

Late closures of MHs, even after revision surgery, have also been documented, suggesting that some MHs might close spontaneously over extended periods. Research indicates that a proportion of patients may benefit from observation when postoperative conditions are stable, and thus avoiding unnecessary surgical interventions [122, 124]. This approach can be particularly relevant in elderly patients or those with multiple comorbidities, where the risk of surgery may outweigh the benefits of intervention.

Cohort studies have reported a range of anatomical closure rates in revision surgeries, from 40% to as high as 75%, depending on the complexity of the case and the specific historical context of each patient’s surgical history [16, 101]. The nuances of MH repair reinforce the need for personalized approaches to treatment, advocating for improved patient selection and optimization of surgical techniques related to FTMH.

***Consensus Statement 8.2:*** *A tailored, patient-specific approach based on previous surgical history and MH characteristics is essential for optimizing revision surgery outcomes in FTMH. [Consensus score: 96.3% (strongly agree: 70.37%; agree: 25.93%)].*

Postoperative visual recovery after re-surgery remains another significant consideration. Various studies reveal that even when anatomical closure is achieved, visual acuity may not always improve correspondingly. This disparity occurs due to multiple factors, including retinal structural integrity and the degree of preoperative macular damage [90, 101]. Understanding the multifactorial nature of visual outcomes is critical for managing patient expectations and refining surgical strategies.

OCT biomarkers play a crucial role in predicting closure and visual prognosis for MHs. Key structural indicators, such as the postoperative restoration of the integrity of the inner segment/outer segment (IS/OS) junction, and external limiting membrane (ELM), significantly correlate with visual outcomes post-surgery. A disrupted IS/OS band is associated with poorer visual recovery, while its restoration suggests better functional results [125, 126]. Additionally, OCT-based preoperative minimum MH diameter and basal diameter, MH indices improve prognostic accuracy [127].

***Consensus Statement 8.3:*** *Postoperative visual outcomes following MH re-surgery depend not only on anatomical closure but also on preoperative retinal integrity and restoration of key OCT biomarkers such as the IS/OS junction and ELM. [Consensus score: 100% (strongly agree: 59.26%; agree: 40.74%)].*

In conclusion, while re-surgery for failed FTMHs poses various challenges, ongoing research and advancements in surgical techniques offer hope for improved outcomes. The application of inverted ILM flaps, careful evaluation of ERMs, and the integration of adjunctive therapies are critical considerations in this complex field. Increased focus on surgical experience and individualized patient care can augment chances of success in these intricate cases, ultimately leading to better anatomical and functional outcomes.

### Emerging technologies and approaches

Minimally invasive techniques in the management of MHs have sparked significant debate in the ophthalmic community. Modifications and advances in microincision vitrectomy systems (MIVS) like dual port cutters and beveled tip cutters enhance safety, and offer faster recovery, while minimizing surgical trauma.

Heads-up 3D visualization systems, in contrast to traditional microscopy, provide real-time, high-definition views of the retina, higher magnification, and better visualization of dye staining. This potentially improves surgical precision by allowing better assessment of intraoperative structures [128]. Additionally, intraoperative OCT guidance helps optimize outcomes during surgery by allowing surgeons to visualize the MH and surrounding tissues in real time, enhancing decision-making and planning [129]. Moreover, the endoillumination power can be reduced to lower than 10% compared to conventional microscopy reducing the risk of light-induced toxicity [130].

Emerging imaging modalities, such as adaptive optics and OCT angiography**,** are generating interest in assessing vascular changes in the retina and understanding their implications for healing and recovery. Although debates around the utility of these advanced techniques continue, their integration into clinical practice may hold some promise for improving MH management strategies.

***Consensus Statement 9.1:*** *The incorporation of MIVS, heads-up 3D visualization, and intraoperative OCT may enhance surgical precision and safety in MH repair. [Consensus score: 74.08% (strongly agree: 25.93%; agree: 48.15%)].*

***Consensus Statement 9.2:*** *While MIVS is definitely beneficial, heads-up 3D visualization is equivocal. Intraoperative OCT definitely does not make much difference. [Consensus score: 66.67% (strongly agree: 25.93%; agree: 40.74%)].*

#### Role of artificial intelligence in MH management

##### Postoperative prognostication using predictive OCT modeling

Recent advances in deep learning have enabled the generation of predictive OCT images that simulate postoperative retinal anatomy. Specifically, generative deep learning models (GDLM) have demonstrated the ability to forecast the restoration of critical biomarkers such as the ELM and ellipsoid zone (EZ), both closely associated with visual recovery after MH surgery. Sharing these simulated outcomes with patients may enhance understanding of surgical goals and benefits, facilitating more informed decision-making. This personalized visual forecasting represents a novel tool in surgical counselling and postoperative expectation management [131].

##### Artificial intelligence (AI)-assisted diagnosis and surgical planning

AI models, particularly convolutional neural networks (CNNs), have shown promise in automatically detecting abnormal retinal features from OCT scans with high accuracy. Rather than classifying full pathologies, recent approaches focus on identifying specific retinal signs related to MHs, thereby providing clinicians with interpretable, actionable insights. Techniques like Grad-CAM visualization further enhance the transparency of these models, allowing ophthalmologists to validate AI decisions visually. While current limitations include the need for large labelled datasets, ongoing improvements in model architecture, and dataset efficiency suggest an expanding role for AI in both diagnosis and preoperative planning for the MH surgery [132–134].

In summary, AI-based tools are increasingly enhancing MH care by enabling accurate postoperative forecasting and aiding in diagnostic interpretation. As these technologies mature, they may significantly improve individualized treatment strategies and support evidence-based clinical decision-making.

***Consensus Statement 9.3:*** *AI, through predictive OCT modelling and feature-specific image analysis, offers promising support in surgical planning, prognosis estimation, and patient counselling for MH management. [Consensus score: 62.96% (strongly agree: 22.22%; agree: 40.74%)].*

## Results

The results of the voting on the consensus statements from each subheading as detailed above are tabulated in Table 2.

**Table 2:** Results of the voting of the consensus statements of macular hole surgery

Section	Consensus statement	C score	Strongly agree	Agree	Neutral	Disagree	Strongly disagree
1. Internal limiting membrane peeling							
1.1	Internal limiting membrane (ILM) peeling is considered a standard procedure in macular hole surgery	**100%**	92.59%	7.41%	0%	0%	0%
1.2	The extent of the ILM peeling plays a crucial role in the surgical management of macular holes, especially for holes larger than 400 μm	**96.3%**	66.67%	29.63%	3.70%	0%	0%
1.3	For larger macular holes and macular holes with the macular hole index or macular hole closure index < 0.5, a 4-disc area of peeling centered on the macular hole would be safer for achieving anatomical success	**88.89%**	37.04%	51.85%	7.41%	3.70%	0%
1.4	Functional outcomes can be marred by micro-scotomata, reduced retinal sensitivity, and decreased vision (although not that common) despite successful structural outcomes and hence judicious, careful and atraumatic peeling of ILM is essential	**92.6%**	55.56%	37.04%	7.41%	0%	0%
2. Vital dye selection							
2.1	Brilliant blue G (BBG) is a good choice of dye for ILM staining due to its effective visualization properties and lower risk of retinal toxicity compared to alternatives such as indocyanine green (ICG) and trypan blue	**88.89%**	48.15%	40.74%	11.11%	0%	0%
2.2	Minimizing dye contact time to approximately 30 s to 1 min is recommended to ensure sufficient ILM staining while reducing the risk of phototoxic effects on the retina	**92.6%**	55.56%	37.04%	3.70%	3.70%	0%
2.3	Reducing intraoperative light intensity to reduce the photo-chemical interaction and toxicity during dye-assisted procedures is essential to prevent phototoxic damage and optimize retinal safety, especially when using dyes with higher toxicity profiles such as ICG	**92.6%**	55.56%	37.04%	7.40%	0%	0%
3. Face-down positioning							
3.1	Face-down positioning remains a widely used postoperative strategy, particularly in cases of large or refractory macular holes, to enhance gas tamponade efficacy and promote anatomical closure	**88.89%**	59.26%	29.63%	7.41%	3.70%	0%
3.2	Emerging evidence suggests that successful macular hole closure may still be achieved without strict adherence to face-down positioning, especially when intraocular gas tamponade is sufficient and tailored to patient-specific factors	**85.18%**	40.74%	44.44%	7.41%	7.41%	0%
3.3	Early postoperative imaging with swept-source optical coherence tomography (OCT) may allow for individualized guidance on face-down positioning, potentially reducing patient burden without compromising surgical outcomes	**85.18%**	33.33%	51.85%	7.41%	7.41%	0%
4. Tamponade selection							
4.1	The selection of endotamponade agents in macular hole surgery should be personalized based on hole size, chronicity, and patient-specific factors	**100%**	66.67%	33.33%	0%	0%	0%
4.2	SF6 gas is an effective tamponade option in routine idiopathic macular hole surgeries, balancing closure rates and recovery time	**81.48%**	40.74%	40.74%	18.52%	0%	0%
4.3	Long-acting gases such as C3F8 improve anatomical success in large macular holes but increase patient burden due to prolonged face-down positioning and cataract risk	**92.59%**	51.85%	40.74%	3.70%	0%	3.7%
4.4	Air tamponade is a viable choice for small or uncomplicated macular holes, despite reduced success rates in larger or chronic cases	70.37%	37.04%	33.33%	22.22%	7.41%	0%
4.5	Silicone oil tamponade should be reserved for recurrent or complex macular holes, due consideration to its long-term complications and the need for a second surgery	**88.89%**	48.15%	40.74%	7.41%	3.70%	0%
5. Surgery timing and indications							
5.1	Early surgical intervention is recommended for larger macular holes or those associated with vitreomacular traction (VMT) to prevent foveal architectural damage and improve visual outcomes	**85.18%**	40.74%	44.44%	11.11%	3.70%	0%
5.2	Small, asymptomatic macular holes (< 250 µm) may be managed conservatively with an initial observation period, provided regular OCT monitoring is in place	62.96%	33.33%	29.63%	18.52%	18.52%	0%
5.3	The presence of VMT is a strong indication for early surgery due to its association with hole progression and retinal damage	**77.78%**	29.63%	48.15%	11.11%	11.11%	0%
5.4	Visual outcomes are generally better when macular hole surgery is performed within 3 months of symptom onset, though individualized timing may still yield favorable results. In general, earlier surgery is recommended	**100%**	59.26%	40.74%	0%	0%	0%
5.5	The decision between immediate surgery and delayed observation should be individualized, taking into account characteristics of the hole, success rate and patient’s visual need and willingness to accept the surgery, etc.	**92.59%**	59.26%	33.33%	3.70%	3.70%	0%
6. Management of special cases							
6.1	Macular hole size > 400 μm is a strong predictor of surgical failure correlating with both poorer anatomical closure rates and diminished visual outcomes	**88.88%**	44.44%	44.44%	7.41%	3.70%	0%
6.2	Prolonged duration of symptoms prior to surgery significantly increases the risk of surgical failure, due to retinal atrophy and fibrotic changes associated with chronic full-thickness macular hole (FTMH)	**92.59%**	62.96%	29.63%	0%	7.41%	0%
6.3	The presence of epiretinal membranes (ERMs) and vitreofoveal attachments are key contributors to surgical failure in macular hole repair, due to increased tangential traction and structural complexity	55.55%	22.22%	33.33%	33.33%	11.11%	0%
6.4	High myopia independently raises the likelihood of unsuccessful macular hole closure, owing to associated vitreoretinal structural abnormalities	**100%**	66.67%	33.33%	0%	0%	0%
6.5	Patient-related factors such as older age, systemic comorbidities (e.g., diabetes, hypertension), prior failed surgery, and poor preoperative visual acuity substantially affect surgical prognosis and visual recovery. While patients with macular hole in eyes with diabetic retinopathy may have poor outcome, diabetes and hypertension themselves may not be poor prognostic indicator	**88.89%**	51.85%	37.04%	3.70%	3.70%	3.70%
7. Adjuvants therapies							
7.1	The inverted ILM flap and translocation techniques significantly improve anatomical closure rates in persistent or macular holes larger than 400 µm	**92.59%**	62.96%	29.63%	3.70%	3.70%	0%
7.2	It is surgically challenging to maintain free ILM graft over the hole. However, the non-inverted free ILM flap technique shows good results and is worth considering but further studies to confirm its validity is warranted	74.08%	37.04%	37.04%	22.22%	3.70%	0%
7.3	Autologous lens capsule flap transplantation is a viable option when ILM tissue is unavailable for grafting	74.08%	25.93%	48.15%	22.22%	3.70%	0%
7.4	Amniotic membrane graft (AMG) is an option to be resorted to if ILM techniques have failed and lens capsular graft harvesting is not possible. They can anatomically close macular holes but often yield suboptimal visual outcomes. Due to limited functional recovery, these techniques are reserved as last-resort options when conventional surgeries fail to achieve hole closure	**96.3%**	37.04%	59.26%	3.70%	0%	0%
7.5	Autologous retinal transplantation (ART) is an option to be resorted to if ILM techniques have failed and lens capsular graft harvesting is not possible. They can anatomically close macular holes but often yield suboptimal visual outcomes. Due to limited functional recovery, these techniques are reserved as last-resort options when conventional surgeries fail to achieve hole closure	**88.88%**	44.44%	44.44%	0%	3.70%	7.41%
7.6	Macular buckle surgery can be an effective strategy, particularly in myopic traction maculopathy or cases requiring axial length reduction. Further studies are warranted	**81.48%**	33.33%	48.15%	14.81%	3.70%	0%
7.7	Low power and mildly intense laser burn applied at the base of the macular hole can be considered as an easy and less manipulative method to achieve successful closure of high myopic macular holes or macular holes ranging from 400–800 µm	33.33%	18.52%	14.81%	22.22%	29.63%	14.81%
8. Visual outcomes and recovery							
8.1	Visual improvement after anatomical closure in revision FTMH surgeries is not guaranteed. However, vision does improve in most cases after anatomical closure though improvement may be suboptimal and quite depends on preoperative retinal integrity	**96.29%**	62.96%	33.33%	3.70%	0%	0%
8.2	A tailored, patient-specific approach based on previous surgical history and macular hole characteristics is essential for optimizing revision surgery outcomes in FTMH	**96.3%**	70.37%	25.93%	0%	3.70%	0%
8.3	Postoperative visual outcomes following macular hole re-surgery depend not only on anatomical closure but also on preoperative retinal integrity and restoration of key OCT biomarkers such as the inner segment/outer segment junction and external limiting membrane	**100%**	59.26%	40.74%	0%	0%	0%
9. Emerging technologies and approaches							
9.1	The incorporation of microincision vitrectomy systems (MIVS), heads-up 3D visualization, and intraoperative OCT may enhance surgical precision and safety in macular hole repair	74.08%	25.93%	48.15%	22.22%	3.70%	0%
9.2	While MIVS is definitely beneficial, heads-up 3D visualization is equivocal. Intraoperative OCT definitely does not make much difference	66.67%	25.93%	40.74%	18.52%	11.11%	3.70%
9.3	Artificial intelligence, through predictive OCT modeling and feature-specific image analysis, offers promising support in surgical planning, prognosis estimation, and patient counseling for macular hole management	62.96%	22.22%	40.74%	29.63%	7.41%	0%

Consensus score (C score) was defined as the value of the summation of the ‘strongly agree’, and ‘agree’ percentages; C score ≥ 75% was considered ‘consensus achieved’ and C score < 75% indicates ‘consensus not reached’. ‘Consensus not achieved’ values are regular plain and underlined. Statements where consensus was achieved have values documented in bold-face

## Discussion

While reviewing the consensus statements on MH surgery, several key issues emerged that merit further discussion. One important aspect is the role of minimum linear diameter (MLD) in surgical decision-making. A recent large study highlighted MLD as a critical determinant of surgical success, particularly for outcomes like MH closure and visual acuity at six months postoperatively [66]. Compared to the traditional view of using 400 μm as a cut-off for large MH, the same study found that success rate of MH closure starts to drop at a threshold of 500 μm (from 97% to 90%) and such nuances would serve as the new benchmark in upcoming studies and discussions [66]. An upcoming study from the same group is expected to refine these thresholds.

The voting results on consensus statements in MH surgery reveal areas of strong agreement, as well as topics where clinical uncertainty persists. Most notably, there was near-unanimous support for ILM peeling as the standard surgical approach (Consensus Statement 1.1, 100% consensus). However, extending the ILM peel to a 4 DD area for larger holes (Consensus Statement 1.3) showed lower consensus (88.89%), reflecting ongoing debates about balancing anatomical success with potential microstructural damage (Consensus Statement 1.4). The discussion around ILM peeling size is also evolving. An IPD meta-analysis of 370 eyes from 5 RCTs found that extending the ILM peel to at least 1 DD radius slightly improved closure rates without negatively impacting visual acuity, especially for holes > 400 μm [39]. This supports our consensus that an adequate extent of peeling is crucial in larger holes.

Intraoperatively, BBG was preferred as a staining dye (Consensus Statement 2.1, 88.89%), while recommendations to limit dye contact time and light intensity (Consensus Statements 2.2 and 2.3) also received high agreement (> 92%), highlighting surgeon concern over phototoxicity.

Postoperative positioning showed mixed responses: face-down positioning was widely endorsed (Consensus Statement 3.1, 88.89%), but the role of alternative positioning strategies (Consensus Statements 3.2 and 3.3) remains debated, likely reflecting recent evidence questioning strict adherence. Regarding face-down positioning, an IPD analysis involving 375 eyes from five RCTs demonstrated that face-down positioning confers a modest but significant benefit in both closure and vision outcomes, particularly for holes > 400 μm. The number needed to treat improved from 15 overall to 12 in this subgroup, indicating a more targeted recommendation for face-down positioning in larger holes [52]. Interestingly, hole chronicity did not influence this relationship, suggesting that size, rather than duration, may be the more critical driver for recommending face-down positioning, a point that aligns with current clinical practice and our consensus statements [66].

Tamponade selection saw strong consensus on personalization (Consensus Statement 4.1, 100%) and using long-acting gases like C3F8 for large holes (Consensus Statement 4.3, 92.59%). Conversely, air tamponade (Consensus Statement 4.4) showed only moderate consensus (70.37%), mirroring concerns about efficacy in larger holes. Special cases, such as holes > 400 µm and prolonged symptoms, consistently reached high agreement (Consensus Statements 6.1 and 6.2). However, managing ERMs and vitreofoveal traction (Consensus Statement 6.3, 55.55%) saw the most divided responses, emphasizing the need for individualized approaches.

There was good agreement overall regarding adjuvant techniques using ILM techniques which are resorted to challenging MH with higher chances of failure. Surgeons agree that amniotic membrane and autologous retinal grafts may be used as last resorts if ILM harvesting is not possible anymore although visual outcomes may be limited. A consensus was also obtained for myopic traction maculopathy, where macular buckle may be a good option.

Collectively, these results underscore both established practices and areas ripe for further research, particularly regarding peel extent, tamponade choice, and adjuvant therapies in complex cases.

## Conclusion

This consensus-building exercise on MH surgery highlights the challenges in surgical decision-making in managing MH. The final voting results highlight the importance of individualized patient factors such as hole size, chronicity, lens status, and preoperative visual acuity in surgical planning and tempering patient’s postoperative expectations. There was strong agreement among the panelists on the value of ILM peeling in enhancing anatomical closure, particularly in cases of larger holes where the risk of surgical failure is more pronounced. Similarly, the consensus statements reflect an appreciation for adjunctive measures, including the inverted ILM flap technique, which has demonstrated improved closure rates in more complex cases. While the evidence around face-down positioning continues to evolve, panelists recognized its selective benefit in large or refractory holes, while also acknowledging patient comfort and individual factors. MH surgery continues to evolve with impressive success rates. However, significant controversies remain regarding optimal surgical techniques, patient management protocols, and outcome assessment. Ongoing research and prospective randomized trials are needed to address these debates and further refine the approach to this common vitreoretinal condition. Our consensus attempted to provide a framework to guide clinicians in the surgical management of MHs, emphasizing a patient-centered, and evidence-based approach that is adaptable as new data continues to emerge.

## Data Availability

All data is available from international medical literature.

## Abbreviations

AAPPO: Academy of Asia–Pacific Professors of Ophthalmology
AMG: Amniotic membrane grafting
APVRS: Asia-Pacific Vitreo-Retina Society
ART: Autologous retinal transplantation
BBG: Brilliant blue G
CNN: Convolutional neural network
DD: Disc diameter
ELM: External limiting membrane
ERM: Epiretinal membrane
EZ: Ellipsoid zone
FDA: Food and Drug Administration
FTMH: Full-thickness macular hole
GDLM: Generative deep learning model
ICG: Indocyanine green
ILM: Internal limiting membrane
IMH: Idiopathic macular hole
IPD: Individual participant data
IPE: International panel of experts
IS/OS: Inner segment/outer segment
IVTS: International Vitreomacular Traction Study
MH: Macular hole
MIVS: Microincision vitrectomy systems
MLD: Minimum linear diameter
OCT: Optical coherence tomography
PFAS: Polyfluoroalkyl substance
PIMS: Positioning in macular hole surgery
RCT: Randomized controlled trial
VMT: Vitreomacular traction

## References

[1] Duker JS, Kaiser PK, Binder S, de Smet MD, Gaudric A, Reichel E, et al. The International Vitreomacular Traction Study Group classification of vitreomacular adhesion, traction, and macular hole. Ophthalmology. 2013;120(12):2611–9. 24053995 10.1016/j.ophtha.2013.07.042

[2] Madi HA, Dinah C, Rees J, Steel DHW. The case mix of patients presenting with full-thickness macular holes and progression before surgery: implications for optimum management. Ophthalmologica. 2015;233(3–4):216–21. 25765054 10.1159/000375378

[3] Shukla SY, Afshar AR, Kiernan DF, Hariprasad SM. Outcomes of chronic macular hole surgical repair. Indian J Ophthalmol. 2014;62(7):795–8. 25116773 10.4103/0301-4738.138302 PMC4152650

[4] Wang J, Rodriguez SH, Xiao J, Luo W, Gonnah R, Shaw L, et al. Full-thickness macular hole closure with topical medical therapy. Retina. 2024;44(3):392–9. 37948745 10.1097/IAE.0000000000003988

[5] Stalmans P, Benz MS, Gandorfer A, Kampik A, Girach A, Pakola S, et al. Enzymatic vitreolysis with ocriplasmin for vitreomacular traction and macular holes. N Engl J Med. 2012;367(7):606–15. 22894573 10.1056/NEJMoa1110823

[6] Scottish Medicines Consortium. Ocriplasmin (Jetrea). https://scottishmedicines.org.uk/medicines-advice/ocriplasmin-jetrea-resubmission-89213/?utm_source=chatgpt.com. Accessed 4 Jun 2025.

[7] Grinton M, Steel DH. Cochrane corner: ocriplasmin—why isn’t it being used more? Eye (Lond). 2019;33(8):1195–7. 30940886 10.1038/s41433-019-0407-1 PMC7005710

[8] Ghoraba H, Rittiphairoj T, Akhavanrezayat A, Karaca I, Matsumiya W, Pham B, et al. Pars plana vitrectomy with internal limiting membrane flap versus pars plana vitrectomy with conventional internal limiting membrane peeling for large macular hole. Cochrane Database Syst Rev. 2023;8:015031. 10.1002/14651858.CD015031.pub2 PMC10558045 37548231

[9] Chen G, Tzekov R, Jiang F, Mao S, Tong Y, Li W. Inverted ILM flap technique versus conventional ILM peeling for idiopathic large macular holes: a meta-analysis of randomized controlled trials. PLoS One. 2020;15(7):e0236431. 32706833 10.1371/journal.pone.0236431 PMC7380636

[10] Khodani M, Bansal P, Narayanan R, Chhablani J. Inverted internal limiting membrane flap technique for very large macular hole. Int J Ophthalmol. 2016;9(8):1230–2. 27588280 10.18240/ijo.2016.08.22 PMC4990591

[11] Ch’ng SW, Elaraoud I, Karl D, Kalogeropoulos D, Lee R, Carreras E. A combination of surgical techniques to repair a giant traumatic macular hole. Case Rep Ophthalmol Med. 2018;2018:7595873. 10.1155/2018/7595873. 30627468 10.1155/2018/7595873 PMC6304587

[12] Deshpande R, Narayanan R. Surgical repair of a giant idiopathic macular hole by inverted internal limiting membrane flap. BMJ Case Rep. 2015;2015:bcr2015210797. 10.1136/bcr-2015-210797. 26025977 10.1136/bcr-2015-210797 PMC4458638

[13] Rezende FA, Ferreira BG, Rampakakis E, Steel DH, Koss MJ, Nawrocka ZA, et al. Surgical classification for large macular hole: based on different surgical techniques results: the CLOSE study group. Int J Retina Vitreous. 2023;30(9):4. 10.1186/s40942-022-00439-4 PMC9885593 36717928

[14] Murphy DC, Al-Zubaidy M, Lois N, Scott N, Steel DH, Macular Hole Duration Study Group. The effect of macular hole duration on surgical outcomes: an individual participant data study of randomized controlled trials. Ophthalmology. 2023;130(2):152–63. 36058348 10.1016/j.ophtha.2022.08.028

[15] Dera AU, Stoll D, Schoeneberger V, Walckling M, Brockmann C, Fuchsluger TA, et al. Anatomical and functional results after vitrectomy with conventional ILM peeling versus inverted ILM flap technique in large full-thickness macular holes. Int J Retina Vitreous. 2023;9(1):68. 37964333 10.1186/s40942-023-00509-1 PMC10644592

[16] Maguire MJ, Steel DH, Yorston D, Hind J, El-Faouri M, Jalil A, et al. Outcome of revision procedures for failed primary macular hole surgery. Retina. 2021;41(7):1389–95. 33315821 10.1097/IAE.0000000000003072

[17] Michalewska Z, Michalewski J, Nawrocki J. Continuous changes in macular morphology after macular hole closure visualized with spectral optical coherence tomography. Graefes Arch Clin Exp Ophthalmol. 2010;248(9):1249–55. 20379735 10.1007/s00417-010-1370-5

[18] Bajdik B, Vajas A, Kemenes G, Fodor M, Surányi É, Takács L. Prediction of long-term visual outcome of idiopathic full-thickness macular hole surgery using optical coherence tomography parameters that estimate potential preoperative photoreceptor damage. Graefes Arch Clin Exp Ophthalmol. 2024;262(10):3181–9. 38717606 10.1007/s00417-024-06500-2 PMC11458759

[19] Mase Y, Matsui Y, Imai K, Imamura K, Irie-Ota A, Chujo S, et al. Preoperative OCT characteristics contributing to prediction of postoperative visual acuity in eyes with macular hole. J Clin Med. 2024;13(16):4826. 39200968 10.3390/jcm13164826 PMC11355252

[20] Lee WH, Jo YJ, Kim JY. Thickness of the macula, retinal nerve fiber layer, and ganglion cell-inner plexiform layer in the macular hole: the repeatability study of spectral-domain optical coherence tomography. Korean J Ophthalmol. 2018;32(6):506–16. 30549475 10.3341/kjo.2018.0030 PMC6288014

[21] Liu P, Sun Y, Dong C, Song D, Jiang Y, Liang J, et al. A new method to predict anatomical outcome after idiopathic macular hole surgery. Graefes Arch Clin Exp Ophthalmol. 2016;254(4):683–8. 26254111 10.1007/s00417-015-3116-x

[22] Alkabes M, Padilla L, Salinas C, Nucci P, Vitale L, Pichi F, et al. Assessment of OCT measurements as prognostic factors in myopic macular hole surgery without foveoschisis. Graefes Arch Clin Exp Ophthalmol. 2013;251(11):2521–7. 23695656 10.1007/s00417-013-2347-y

[23] Wakely L, Rahman R, Stephenson J. A comparison of several methods of macular hole measurement using optical coherence tomography, and their value in predicting anatomical and visual outcomes. Br J Ophthalmol. 2012;96(7):1003–7. 22611137 10.1136/bjophthalmol-2011-301287

[24] Ruamviboonsuk P, Ng DSC, Chaikitmongkol V, Chang A, Chen SJ, Chen Y, et al. Consensus and guidelines on diagnosis and management of polypoidal choroidal vasculopathy (PCV) from the Asia-Pacific Vitreo-retina Society (APVRS). Asia Pac J Ophthalmol (Phila). 2025;14(1):100144. 39824255 10.1016/j.apjo.2025.100144

[25] Richards K, Kadakia A, Wykoff CC, Major JC, Wong TP, Chen E, et al. Management of large full-thickness macular holes: long-term outcomes of internal limiting membrane flaps and internal limiting membrane peels. Retina. 2024;44(7):1165–70. 38900578 10.1097/IAE.0000000000004099

[26] Brooks HL. Macular hole surgery with and without internal limiting membrane peeling. Ophthalmology. 2000;107(10):1939–48. 11013203 10.1016/s0161-6420(00)00331-6

[27] Spiteri Cornish K, Lois N, Scott N, Burr J, Cook J, Boachie C, et al. Vitrectomy with internal limiting membrane (ILM) peeling versus vitrectomy with no peeling for idiopathic full-thickness macular hole (FTMH). Cochrane Database Syst Rev. 2013;6:CD009306. 10.1002/14651858.CD009306.pub2 PMC12551316 23740611

[28] Michalewska Z, Michalewski J, Adelman RA, Nawrocki J. Inverted internal limiting membrane flap technique for large macular holes. Ophthalmology. 2010;117(10):2018–25. 20541263 10.1016/j.ophtha.2010.02.011

[29] Michalewska Z, Michalewski J, Dulczewska-Cichecka K, Adelman RA, Nawrocki J. Temporal inverted internal limiting membrane flap technique versus classic inverted internal limiting membrane flap technique: a comparative study. Retina. 2015;35(9):1844–50. 25946691 10.1097/IAE.0000000000000555

[30] Nie ZT, Liu BS, Wang Y, Chen Q, Wei JT, Yang M, et al. Negative effects of enlarging internal limiting membrane peeling for idiopathic macular hole surgery. Int J Ophthalmol. 2022;15(11):1806–13. 36404972 10.18240/ijo.2022.11.11 PMC9631185

[31] Goel N, Shukla G. Long-term follow up of en face optical coherence tomography of the inner retinal surface following internal limiting membrane peeling for idiopathic macular holes. Int Ophthalmol. 2021;41(3):1003–10. 33200392 10.1007/s10792-020-01657-1

[32] Liu J, Chen Y, Wang S, Zhang X, Zhao P. Evaluating inner retinal dimples after inner limiting membrane removal using multimodal imaging of optical coherence tomography. BMC Ophthalmol. 2018;18(1):155. 29945560 10.1186/s12886-018-0828-9 PMC6020371

[33] Tadayoni R, Svorenova I, Erginay A, Gaudric A, Massin P. Decreased retinal sensitivity after internal limiting membrane peeling for macular hole surgery. Br J Ophthalmol. 2012;96(12):1513–6. 23077227 10.1136/bjophthalmol-2012-302035 PMC3512349

[34] Terasaki H, Miyake Y, Nomura R, Piao CH, Hori K, Niwa T, et al. Focal macular ERGs in eyes after removal of macular ILM during macular hole surgery. Invest Ophthalmol Vis Sci. 2001;42(1):229–34. 11133873

[35] Tadayoni R, Paques M, Massin P, Mouki-Benani S, Mikol J, Gaudric A. Dissociated optic nerve fiber layer appearance of the fundus after idiopathic epiretinal membrane removal. Ophthalmology. 2001;108(12):2279–83. 11733271 10.1016/s0161-6420(01)00856-9

[36] Morescalchi F, Costagliola C, Gambicorti E, Duse S, Romano MR, Semeraro F. Controversies over the role of internal limiting membrane peeling during vitrectomy in macular hole surgery. Surv Ophthalmol. 2017;62(1):58–69. 27491476 10.1016/j.survophthal.2016.07.003

[37] Tadayoni R, Gaudric A, Haouchine B, Massin P. Relationship between macular hole size and the potential benefit of internal limiting membrane peeling. Br J Ophthalmol. 2006;90(10):1239–41. 16809385 10.1136/bjo.2006.091777 PMC1857449

[38] Wiedemann P. How internal limiting membrane peeling revolutionized macular surgery in the last three decades. Int J Ophthalmol. 2023;16(6):837–40. 37332552 10.18240/ijo.2023.06.01 PMC10250952

[39] Teh BL, Li Y, Nanji K, Phillips M, Chaudhary V, Steel DH, et al. Internal limiting membrane peel size and macular hole surgery outcome: a systematic review and individual participant data study of randomized controlled trials. Eye (Lond). 2025;39(7):1406–13. 39922971 10.1038/s41433-025-03666-9 PMC12044072

[40] Bae K, Kang SW, Kim JH, Kim SJ, Kim JM, Yoon JM. Extent of internal limiting membrane peeling and its impact on macular hole surgery outcomes: a randomized trial. Am J Ophthalmol. 2016;169:179–88. 27393470 10.1016/j.ajo.2016.06.041

[41] Khodabande A, Mahmoudi A, Faghihi H, Bazvand F, Ebrahimi E, Riazi-Esfahani H. Outcomes of idiopathic full-thickness macular hole surgery: comparing two different ILM peeling sizes. J Ophthalmol. 2020;18(2020):1619450. 10.1155/2020/1619450 PMC7450298 32908679

[42] Yao Y, Qu J, Dong C, Li X, Liang J, Yin H, et al. The impact of extent of internal limiting membrane peeling on anatomical outcomes of macular hole surgery: results of a 54-week randomized clinical trial. Acta Ophthalmol. 2019;97(3):303–12. 30187641 10.1111/aos.13853 PMC6585771

[43] Kusuhara S, Teraoka Escaño MF, Fujii S, Nakanishi Y, Tamura Y, Nagai A, et al. Prediction of postoperative visual outcome based on hole configuration by optical coherence tomography in eyes with idiopathic macular holes. Am J Ophthalmol. 2004;138(5):709–16. 15531303 10.1016/j.ajo.2004.04.063

[44] Brockmann T, Steger C, Westermann M, Nietzsche S, Koenigsdoerffer E, Strobel J, et al. Ultrastructure of the membrana limitans interna after dye-assisted membrane peeling. Ophthalmologica. 2011;226(4):228–33. 21893971 10.1159/000331218

[45] Naithani P, Vashisht N, Khanduja S, Sinha S, Garg S. Brilliant blue G-assisted peeling of the internal limiting membrane in macular hole surgery. Indian J Ophthalmol. 2011;59(2):158–60. 21350290 10.4103/0301-4738.77047 PMC3116549

[46] Steel DH, Dinah C, Madi HA, White K, Rees J. The staining pattern of brilliant blue G during macular hole surgery: a clinicopathologic study. Invest Ophthalmol Vis Sci. 2014;55(9):5924–31. 25125604 10.1167/iovs.14-14809

[47] Gandorfer A, Haritoglou C, Gandorfer A, Kampik A. Retinal damage from indocyanine green in experimental macular surgery. Invest Ophthalmol Vis Sci. 2003;44(1):316–23. 12506091 10.1167/iovs.02-0545

[48] Lee KL, Dean S, Guest S. A comparison of outcomes after indocyanine green and trypan blue assisted internal limiting membrane peeling during macular hole surgery. Br J Ophthalmol. 2005;89(4):420–4. 15774917 10.1136/bjo.2004.049684 PMC1772609

[49] Rodrigues EB, Maia M, Penha FM, Dib E, Melo GB, Maia A, et al. Staining properties of brilliant blue depending on different incubation times and solvents in humans. Ophthalmologica. 2013;230(Suppl 2):68–72. 24022721 10.1159/000353872

[50] Shukla D, Kalliath J, Patwardhan A, Kannan NB, Thayyil SB. A preliminary study of Heavy Brilliant Blue G for internal limiting membrane staining in macular hole surgery. Indian J Ophthalmol. 2012;60(6):531–4. 23202392 10.4103/0301-4738.103786 PMC3545130

[51] Hu Z, Xie P, Ding Y, Zheng X, Yuan D, Liu Q. Face-down or no face-down posturing following macular hole surgery: a meta-analysis. Acta Ophthalmol. 2016;94(4):326–33. 26385613 10.1111/aos.12844

[52] Raimondi R, Tzoumas N, Toh S, Sarohia GS, Phillips MR, Chaudhary V, et al. Facedown positioning in macular hole surgery: a systematic review and individual participant data meta-analysis. Ophthalmology. 2025;132(2):194–205. 39147105 10.1016/j.ophtha.2024.08.012

[53] Pasu S, Bell L, Zenasni Z, Lanz D, Simmonds IA, Thompson A, et al. Facedown positioning following surgery for large full-thickness macular hole: a multicenter randomized clinical trial. JAMA Ophthalmol. 2020;138(7):725–30. 32379288 10.1001/jamaophthalmol.2020.0987 PMC7206530

[54] Casini G, Loiudice P, De Cillà S, Radice P, Nardi M. Sulfur hexafluoride (SF6) versus perfluoropropane (C3F8) tamponade and short term face-down position for macular hole repair: a randomized prospective study. Int J Retina Vitreous. 2016;2:10. 27847628 10.1186/s40942-016-0036-9 PMC5088452

[55] Xirou T, Theodossiadis PG, Apostolopoulos M, Kabanarou SA, Feretis E, Ladas ID, et al. Macular hole surgery with short-acting gas and short-duration face-down positioning. Clin Ophthalmol. 2012;6:1107–12. 22973086 10.2147/OPTH.S32077 PMC3422152

[56] Sato M, Iwase T. Swept source-optical coherence tomography-guided facedown posturing to minimize treatment burden and maximize outcome after macular hole surgery. J Clin Med. 2023;12(16):5282. 37629324 10.3390/jcm12165282 PMC10455272

[57] Isomae T, Sato Y, Shimada H. Shortening the duration of prone positioning after macular hole surgery- comparison between 1-week and 1-day prone positioning. Jpn J Ophthalmol. 2002;46(1):84–8. 11853720 10.1016/s0021-5155(01)00468-3

[58] Krohn J. Duration of face-down positioning after macular hole surgery: a comparison between 1 week and 3 days. Acta Ophthalmol Scand. 2005;83(3):289–92. 15948778 10.1111/j.1600-0420.2005.00462.x

[59] Almeida DRP, Wong J, Belliveau M, Rayat J, Gale J. Anatomical and visual outcomes of macular hole surgery with short-duration 3-day face-down positioning. Retina. 2012;32(3):506–10. 22392092 10.1097/IAE.0b013e3182219abd

[60] Ellis JD, Malik TY, Taubert MA, Barr A, Baines PS. Surgery for full-thickness macular holes with short-duration prone posturing: results of a pilot study. Eye (Lond). 2000;14(Pt 3A):307–12. 11026990 10.1038/eye.2000.78

[61] Kelly NE, Wendel RT. Vitreous surgery for idiopathic macular holes. Results of a pilot study. Arch Ophthalmol. 1991;109(5):654–9. 2025167 10.1001/archopht.1991.01080050068031

[62] Alberti M, la Cour M. Gas-foveal contact: a new approach to evaluating positioning regimens in macular hole surgery. Retina. 2018;38(5):913–21. 28463904 10.1097/IAE.0000000000001654

[63] Steel DHW, Parkes C, Papastavrou VT, Avery PJ, El-Ghrably IA, Habib MS, et al. Predicting macular hole closure with ocriplasmin based on spectral domain optical coherence tomography. Eye (Lond). 2016;30(5):740–5. 26965018 10.1038/eye.2016.42 PMC4869143

[64] Zhang Y, Chen X, Hong L, Yan Y, Zeng M, Huang Z, et al. Facedown positioning after vitrectomy will not facilitate macular hole closure based on swept-source optical coherence tomography imaging in gas-filled eyes: a prospective, randomized comparative interventional study. Retina. 2019;39(12):2353–9. 30204729 10.1097/IAE.0000000000002325

[65] Roth DB, Smiddy WE, Feuer W. Vitreous surgery for chronic macular holes. Ophthalmology. 1997;104(12):2047–52. 9400764 10.1016/s0161-6420(97)30060-8

[66] Steel DH, Donachie PHJ, Aylward GW, Laidlaw DA, Williamson TH, Yorston D, et al. Factors affecting anatomical and visual outcome after macular hole surgery: findings from a large prospective UK cohort. Eye (Lond). 2021;35(1):316–25. 32231259 10.1038/s41433-020-0844-x PMC7852599

[67] Stec LA, Ross RD, Williams GA, Trese MT, Margherio RR, Cox MS. Vitrectomy for chronic macular holes. Retina. 2004;24(3):341–7. 15187653 10.1097/00006982-200406000-00001

[68] Dervenis N, Dervenis P, Sandinha T, Murphy DC, Steel DH. Intraocular tamponade choice with vitrectomy and internal limiting membrane peeling for idiopathic macular hole: a systematic review and meta-analysis. Ophthalmol Retina. 2022;6(6):457–68. 35144020 10.1016/j.oret.2022.01.023

[69] Chen X, Yao Y, Hao X, Liu X, Liu T. A comparative study of vitrectomy combined with internal limiting membrane peeling for the treatment of idiopathic macular hole with air or C3F8 intraocular tamponade. J Ophthalmol. 2018;2018:1672501. 30057803 10.1155/2018/1672501 PMC6051092

[70] Briand S, Chalifoux E, Tourville E, Bourgault S, Caissie M, Tardif Y, et al. Prospective randomized trial: outcomes of SF₆ versus C₃F₈ in macular hole surgery. Can J Ophthalmol. 2015;50(2):95–100. 25863847 10.1016/j.jcjo.2014.12.006

[71] Forsaa VA, Krohn J. Air tamponade combined with nonsupine positioning in macular hole surgery for pseudophakic eyes. Retina. 2017;37(9):1750–6. 27902639 10.1097/IAE.0000000000001413

[72] Tao J, Chen H, Chen Y, Yu J, Xu J, Mao J, et al. Efficacy of air tamponade treatment of idiopathic macular holes of different diameters and of follow-up intravitreal air tamponade for persistent holes. Retina. 2022;42(5):877–82. 34954776 10.1097/IAE.0000000000003394

[73] Cillino S, Cillino G, Ferraro LL, Casuccio A. Treatment of persistently open macular holes with heavy silicone oil (Densiron 68) versus C2F6. A prospective randomized study. Retina. 2016;36(4):688–94. 26418444 10.1097/IAE.0000000000000781

[74] Nowroozzadeh MH, Ashraf H, Zadmehr M, Farvardin M. Outcomes of light silicone oil tamponade for failed idiopathic macular hole surgery. J Ophthalmic Vis Res. 2018;13(2):130–7. 29719640 10.4103/jovr.jovr_22_17 PMC5905305

[75] Lai JC, Stinnett SS, McCuen BW. Comparison of silicone oil versus gas tamponade in the treatment of idiopathic full-thickness macular hole. Ophthalmology. 2003;110(6):1170–4. 12799243 10.1016/S0161-6420(03)00264-1

[76] Hong J, Deng SX, Xu J. Enzymatic vitreolysis with ocriplasmin for vitreomacular traction and macular holes. N Engl J Med. 2012;367(21):2053; author reply 2054. 23171110 10.1056/NEJMc1211068

[77] Tew TB, Chen TC, Yang CH, Yang CM. Vitreomacular changes after intravitreal gas injection for idiopathic impending or early macular hole: an optical coherence tomography study. Ophthalmologica. 2018;239(1):1–10. 28954267 10.1159/000478666

[78] Neubauer J, Gelisken F, Ozturk T, Bartz-Schmidt KU, Dimopoulos S. The time course of spontaneous closure of idiopathic full-thickness macular holes. Graefes Arch Clin Exp Ophthalmol. 2024;262(9):2859–65. 38587655 10.1007/s00417-024-06465-2 PMC11377478

[79] Stalmans P. A retrospective cohort study in patients with tractional diseases of the vitreomacular interface (ReCoVit). Graefes Arch Clin Exp Ophthalmol. 2016;254(4):617–28. 26899900 10.1007/s00417-016-3294-1 PMC4799804

[80] Toumi E, Guindolet D, Bonnin S, Bruneau S, Leflot M, Duvillier A, et al. Visual outcomes after spontaneous and surgical closure of small idiopathic macular holes: a comparative study. Ophthalmologica. 2025;248(1):22–8. 39602900 10.1159/000541057

[81] Budoff G, Bhagat N, Zarbin MA. Traumatic macular hole: diagnosis, natural history, and management. J Ophthalmol. 2019;19(2019):5837832. 10.1155/2019/5837832 PMC6444256 31016038

[82] De Clerck I, Bivort J, Van Calster J, Stalmans P. A retrospective study on the outcomes of pneumatic vitreolysis for the treatment of vitreomacular traction. Retina. 2025;45(3):420–5. 39486050 10.1097/IAE.0000000000004319

[83] Patel R, Gopalakrishnan M, Giridhar A. Timing and outcome of surgery for persistent macular hole. Retina. 2019;39(2):314–8. 29135801 10.1097/IAE.0000000000001939

[84] Moussa G, Jalil A, Patton N, Sabatino F, Jasani K, Dhawahir-Scala F, et al. Prediction of macular hole size progression based on baseline optical coherence tomography findings. Retina. 2023;43(3):464–71. 36730582 10.1097/IAE.0000000000003680

[85] Panos GD, Poyser O, Sarwar H, Kumudhan D, Orr G, Zaman A, et al. The impact of the COVID-19 pandemic and lockdown on macular hole surgery provision and surgical outcomes: a single-centre experience. J Clin Med. 2022;11(13):3678. 35806963 10.3390/jcm11133678 PMC9267484

[86] Jaycock PD, Bunce C, Xing W, Thomas D, Poon W, Gazzard G, et al. Outcomes of macular hole surgery: implications for surgical management and clinical governance. Eye (Lond). 2005;19(8):879–84. 15389276 10.1038/sj.eye.6701679

[87] Willis AW, Garcia-Cosio JF. Macular hole surgery. Comparison of longstanding versus recent macular holes. Ophthalmology. 1996;103(11):1811–4. 8942875 10.1016/s0161-6420(96)30422-3

[88] Kokame GT, Johnson MW, Lim J, Flynn HW, de Carlo T, Yannuzzi N, et al. Closure of full-thickness macular holes associated with macular edema with medical therapy. Ophthalmologica. 2022;245(2):179–86. 34182564 10.1159/000516018

[89] Chun JW, Kim CH, Kim JY, Oh HS, Kim SH, Kwon OW, et al. Prevalence and progression of stage 0 macular hole in fellow eyes of patients with idiopathic full-thickness macular hole. Korean J Ophthalmol. 2021;35(2):107–11. 33845555 10.3341/kjo.2020.0078 PMC8046619

[90] Yang JM, Choi SU, Kim YJ, Kim R, Yon DK, Lee SW, et al. Association between epiretinal membrane, epiretinal proliferation, and prognosis of full-thickness macular hole closure. Retina. 2022;42(1):46–54. 34267114 10.1097/IAE.0000000000003262

[91] He HL, Liu YX, Chen XY, Ling SG, Qi Y, Xiong Y, et al. Fundus tessellated density of pathologic myopia. Asia Pac J Ophthalmol (Phila). 2023;12(6):604–13. 38079255 10.1097/APO.0000000000000642

[92] Jonas JB, Panda-Jonas S, Dong L, Jonas RA. Clinical and anatomical features of myopia. Asia Pac J Ophthalmol (Phila). 2024;13(6):100114. 39622437 10.1016/j.apjo.2024.100114

[93] Wu TT, Hou TY, Peng KL, Kung YH. Inverted flap technique versus internal limiting membrane insertion for macular hole in eyes with extremely high myopia. BMC Ophthalmol. 2024;24(1):286. 39009984 10.1186/s12886-024-03566-8 PMC11251341

[94] Wu AL, Ling KP, Chuang LH, Chen KJ, Chen YP, Yeung L, et al. Treatment of macular hole retinal detachment with macular plug in highly myopic eyes: three-year results. Acta Ophthalmol. 2020;98(7):e839–47. 32243725 10.1111/aos.14418

[95] Wu TT, Kung YH, Chang CY, Chang SP. Surgical outcomes in eyes with extremely high myopia for macular hole without retinal detachment. Retina. 2018;38(10):2051–5. 28796147 10.1097/IAE.0000000000001806

[96] Wong SC, Neuwelt MD, Trese MT. Delayed closure of paediatric macular hole in Coats’ disease. Acta Ophthalmol. 2012;90(4):e326-327. 21914147 10.1111/j.1755-3768.2011.02244.x

[97] Brennan N, Reekie I, Khawaja AP, Georgakarakos N, Ezra E. Vitrectomy, inner limiting membrane peel, and gas tamponade in the management of traumatic pediatric macular holes: a case series of 13 patients. Ophthalmologica. 2017;238(3):119–23. 28768260 10.1159/000477177

[98] Lorenzi U, Mehech J, Caporossi T, Romano MR, De Fazio R, Parrat E, et al. A retrospective, multicenter study on the management of macular holes without residual internal limiting membrane: the refractory macular hole (ReMaHo) study. Graefes Arch Clin Exp Ophthalmol. 2022;260(12):3837–45. 35790571 10.1007/s00417-022-05739-x PMC9666308

[99] Yilmaz S, Yildiz AM, Avci R. A novel surgical technique for giant and refractory macular holes: autologous tenon capsule plug. Retin Cases Brief Rep. 2024;1:1–9. 10.1097/ICB.0000000000001585. 10.1097/ICB.0000000000001585 39330766

[100] Pellegrini M, Mura M, Yu AC, Spena R, Ruzza A, Ponzin D, et al. Descemet membrane epiretinal graft for refractory full-thickness macular hole. Ophthalmol Retina. 2024;8(6):611–3. 38494116 10.1016/j.oret.2024.03.011

[101] Nawrocka ZA, Nawrocki J. Predictive factors of surgical success with the inverted internal limiting membrane flap technique. Retina. 2024;44(3):400–5. 37948738 10.1097/IAE.0000000000003986

[102] Pires J, Nadal J, Gomes NL. Internal limiting membrane translocation for refractory macular holes. Br J Ophthalmol. 2017;101(3):377–82. 27146153 10.1136/bjophthalmol-2015-308299

[103] Gekka T, Watanabe A, Ohkuma Y, Arai K, Watanabe T, Tsuzuki A, et al. Pedicle internal limiting membrane transposition flap technique for refractory macular hole. Ophthalmic Surg Lasers Imaging Retina. 2015;46(10):1045–6. 26599248 10.3928/23258160-20151027-10

[104] Rossi T, Trillo C, Ripandelli G. Autologous internal limiting membrane transplant for recurrent idiopathic macular holes. Eur J Ophthalmol. 2020;11:1120672120906391. 10.1177/1120672120906391 32043368

[105] Fung NSK, Mak AKH, Yiu R, Wong IYH, Lam WC. Treatment of large, chronic and persistent macular hole with internal limiting membrane transposition and tuck technique. Int J Retina Vitreous. 2020;6(1):3. 32180996 10.1186/s40942-019-0206-7 PMC7063789

[106] Tian T, Jiao D, Zhang X, Wang M, Guo S, Lyu J, et al. Non-inverted and single-layer “plastic bag” ILM flap novel technique to treat large macular holes. Asia-Pac J Ophthalmol (Phila). 2025;21:100164. 10.1016/j.apjo.2025.100164 39988090

[107] Peng J, Chen C, Zhang H, Zhang L, Liu J, Ren J, et al. Long-term surgical outcomes of lens capsular flap transplantation in the management of refractory macular hole. Retina. 2021;41(4):726–34. 32732611 10.1097/IAE.0000000000002922

[108] Peng J, Chen C, Jin H, Zhang H, Zhao P. Autologous lens capsular flap transplantation combined with autologous blood application in the management of refractory macular hole. Retina. 2018;38(11):2177–83. 29045320 10.1097/IAE.0000000000001830

[109] Chen SN, Yang CM. Lens capsular flap transplantation in the management of refractory macular hole from multiple etiologies. Retina. 2016;36(1):163–70. 26200509 10.1097/IAE.0000000000000674

[110] Caporossi T, Governatori L, Verdina T, Rizzo S. Human amniotic membrane for failed macular hole. A case of initial unsuccessful outcome that resolved after amniotic membrane exchange. Eur J Ophthalmol. 2021. 10.1177/11206721211058996. 34779681 10.1177/11206721211058996

[111] Caporossi T, Pacini B, De Angelis L, Barca F, Peiretti E, Rizzo S. Human amniotic membrane to close recurrent, high myopic macular holes in pathologic myopia with axial length of ≥30 mm. Retina. 2020;40(10):1946–54. 31868775 10.1097/IAE.0000000000002699

[112] Grewal DS, Charles S, Parolini B, Kadonosono K, Mahmoud TH. Autologous retinal transplant for refractory macular holes: multicenter international collaborative study group. Ophthalmology. 2019;126(10):1399–408. 30711606 10.1016/j.ophtha.2019.01.027

[113] Hanai M, Amaral DC, Jacometti R, Aguiar EHC, Gomes FC, Cyrino LG, et al. Large macular hole and autologous retinal transplantation: a systematic review and meta-analysis. Int J Retina Vitreous. 2024;10(1):56. 39175026 10.1186/s40942-024-00573-1 PMC11340077

[114] Grewal DS, Mahmoud TH. Autologous neurosensory retinal free flap for closure of refractory myopic macular holes. JAMA Ophthalmol. 2016;134(2):229–30. 26720054 10.1001/jamaophthalmol.2015.5237

[115] Charles S, Randolph JC, Neekhra A, Salisbury CD, Littlejohn N, Calzada JI. Arcuate retinotomy for the repair of large macular holes. Ophthalmic Surg Lasers Imaging Retina. 2013;44(1):69–72. 23418735 10.3928/23258160-20121221-15

[116] Karacorlu M, Sayman Muslubas I, Hocaoglu M, Arf S, Ersoz MG. Double arcuate relaxing retinotomy for a large macular hole. Retin Cases Brief Rep. 2019;13(2):167–70. 28221260 10.1097/ICB.0000000000000551

[117] Serhan HA, Ahmed A, Chaudhry M, Nadeem ZA, Ahmed F, Kamal UH, et al. Macular buckling for myopic traction maculopathy: a comprehensive systematic review and meta-analysis. Am J Ophthalmol. 2025;270:25–34. 39424027 10.1016/j.ajo.2024.10.008

[118] Ma J, Li H, Ding X, Tanumiharjo S, Lu L. Effectiveness of combined macular buckle under direct vision and vitrectomy with ILM peeling in refractory macular hole retinal detachment with extreme high axial myopia: a 24-month comparative study. Br J Ophthalmol. 2017;101(10):1386–94. 28292775 10.1136/bjophthalmol-2016-310123 PMC5629954

[119] Parolini B, Matello V, Rosales-Padrón JF. Combined surgical approach for repair of refractory macular hole in myopic traction maculopathy. J Vitreoretin Dis. 2024;30:24741264241293908. 10.1177/24741264241293908 PMC11556390 39539819

[120] Parolini B, Frisina R, Pinackatt S, Gasparotti R, Gatti E, Baldi A, et al. Indications and results of a new L-shaped macular buckle to support a posterior staphyloma in high myopia. Retina. 2015;35(12):2469–82. 26079474 10.1097/IAE.0000000000000613

[121] Radke N, Liu S, Lam DSC. Re: Internal limiting membrane (ILM) transplantation for unclosed and large macular holes (MH). Graefes Arch Clin Exp Ophthalmol. 2017;255(5):1047–8. 28353012 10.1007/s00417-017-3628-7

[122] Lee J, Nguyen VQ, Doss MK, Eller AW. Spontaneous closure of a chronic full thickness macular hole after failed surgery. Am J Ophthalmol Case Rep. 2018;13:59–61. 30582073 10.1016/j.ajoc.2018.12.006 PMC6292999

[123] Gonzalez-Cortes JH, Toledo-Negrete JJ, Bages-Rousselon Y, de Alba-Castilla MA, Mohamed-Hamsho J. Spontaneous closure of simultaneous idiopathic macular holes documented by spectral-domain optical coherence tomography. Retin Cases Brief Rep. 2021;15(1):27–30. 29847534 10.1097/ICB.0000000000000749

[124] Parikh PD, Day Ghafoori S. Multiple late recurrences of macular hole after vitrectomy and episode of spontaneous closure in a single eye. Retin Cases Brief Rep. 2021;15(5):635–9. 30913122 10.1097/ICB.0000000000000870

[125] Ooka E, Mitamura Y, Baba T, Kitahashi M, Oshitari T, Yamamoto S. Foveal microstructure on spectral-domain optical coherence tomographic images and visual function after macular hole surgery. Am J Ophthalmol. 2011;152(2):283-290.e1. 21669402 10.1016/j.ajo.2011.02.001

[126] Kitao M, Wakabayashi T, Nishida K, Sakaguchi H, Nishida K. Long-term reconstruction of foveal microstructure and visual acuity after idiopathic macular hole repair: Three-year follow-up study. Br J Ophthalmol. 2019;103(2):238–44. 29632001 10.1136/bjophthalmol-2017-311689

[127] Ullrich S, Haritoglou C, Gass C, Schaumberger M, Ulbig MW, Kampik A. Macular hole size as a prognostic factor in macular hole surgery. Br J Ophthalmol. 2002;86(4):390–3. 11914205 10.1136/bjo.86.4.390 PMC1771090

[128] Palácios RM, Kayat KV, Farah ME, Devin F. Heads-up digitally assisted surgical viewing with intraoperative optical coherence tomography for myopic schisis repair. J Ophthalmic Vis Res. 2021;16(1):127–30. 33520136 10.18502/jovr.v16i1.8259 PMC7841277

[129] Kunikata H, Nakazawa T. Intraoperative optical coherence tomography-assisted 27-gauge vitrectomy in eyes with vitreoretinal diseases. Case Rep Ophthalmol. 2015;6(2):216–22. 26265908 10.1159/000437014 PMC4519593

[130] Horigome Y, Iwashita Y, Hirono K, Inoue T, Konno A, Kadonosono K, et al. Evaluation of the retinal hazard with 3D digitally assisted visualization system and conventional microscope in macular surgeries. Retina. 2022;42(12):2301–6. 36053901 10.1097/IAE.0000000000003621

[131] Kwon HJ, Heo J, Park SH, Park SW, Byon I. Accuracy of generative deep learning model for macular anatomy prediction from optical coherence tomography images in macular hole surgery. Sci Rep. 2024;14(1):6913. 38519532 10.1038/s41598-024-57562-5 PMC10959933

[132] Leandro I, Lorenzo B, Aleksandar M, Dario M, Rosa G, Agostino A, et al. OCT-based deep-learning models for the identification of retinal key signs. Sci Rep. 2023;13(1):14628. 37670066 10.1038/s41598-023-41362-4 PMC10480174

[133] Kucukgoz B, Zou K, Murphy DC, Steel DH, Obara B, Fu H. Uncertainty-aware regression model to predict post-operative visual acuity in patients with macular holes. Comput Med Imaging Graph. 2025;119:102461. 39615266 10.1016/j.compmedimag.2024.102461

[134] Kucukgoz B, Yapici MM, Murphy DC, Spowart E, Steel DH, Obara B. Deep learning using preoperative optical coherence tomography images to predict visual acuity following surgery for idiopathic full-thickness macular holes. IEEE Access. 2024;12:32911–26.

